# Keratinocyte-specific deletion of SHARPIN induces atopic dermatitis-like inflammation in mice

**DOI:** 10.1371/journal.pone.0235295

**Published:** 2020-07-20

**Authors:** John P. Sundberg, C. Herbert Pratt, Leslie P. Goodwin, Kathleen A. Silva, Victoria E. Kennedy, Christopher S. Potter, Anisa Dunham, Beth A. Sundberg, Harm HogenEsch

**Affiliations:** 1 The Jackson Laboratory, Bar Harbor, ME, United States of America; 2 Department of Comparative Pathobiology, College of Veterinary Medicine, Purdue University, West Lafayette, IN, United States of America; INSERM, FRANCE

## Abstract

Spontaneous mutations in the SHANK-associated RH domain interacting protein (*Sharpin*) resulted in a severe autoinflammatory type of chronic proliferative dermatitis, inflammation in other organs, and lymphoid organ defects. To determine whether cell-type restricted loss of *Sharpin* causes similar lesions, a conditional null mutant was created. Ubiquitously expressing *cre*-recombinase recapitulated the phenotype seen in spontaneous mutant mice. Limiting expression to keratinocytes (using a *Krt14-cre*) induced a chronic eosinophilic dermatitis, but no inflammation in other organs or lymphoid organ defects. The dermatitis was associated with a markedly increased concentration of serum IgE and IL18. Crosses with *S100a4-cre* resulted in milder skin lesions and moderate to severe arthritis. This conditional null mutant will enable more detailed studies on the role of SHARPIN in regulating NFkB and inflammation, while the *Krt14-Sharpin*^*-/-*^ provides a new model to study atopic dermatitis.

## Introduction

Spontaneous mutations in SHANK-associated RH domain interacting protein (*Sharpin*) result in a chronic proliferative dermatitis (allele symbol: *cpdm*) phenotype in two different strains of laboratory mice (C57BL/KaLawRij-*Sharpin*^*cpdm*^/RijSunJ and CBy.OcB3/Dem-*Sharpin*^*cpdm-Dem*^) [1, 2]. In the *Sharpin*^*cpdm*^ mutant mice, a single base pair deletion was observed resulting in a shift of the open reading frame predicted to cause an early stop codon beginning at position 624 (numbered sequence is based on Ensembl cDNA transcript ENSMUST00000023211). The *Sharpin*^*cpdm-Dem*^ mutants there was a C to A transition at position 434 in Exon 1 followed by a 14-bp deletion where the 14-bp deletion disrupted the reading frame resulting in an early stop codon. Western blot analysis confirmed loss of SHARPIN protein resulting in both alleles being null mutations [2].

In both mutant mouse strains, homozygous mutants develop a severe, chronic, autoinflammatory disease, affecting multiple organs but especially the skin, where it is typified by progressive epidermal hyperplasia, marked apoptosis of keratinocytes, and a mixed, primarily eosinophilic, dermal inflammation. Homozygous mutant mice exhibit multi-organ eosinophilic inflammation, defective T_H_1 cytokine production, abnormal T cell development, diminished immunoglobulin isotype switching, splenomegaly, early regression of Peyer’s patches, and an absence of B cell follicles, follicular dendritic cells, and germinal centers in secondary lymph organs [3–5]. This phenotype is consistent in both spontaneous null alleles; however, CBy.OcB3/Dem-*Sharpin*^*cpdm-Dem*^ mice have a more rapid onset of symptoms and a shorter lifespan, reflecting strain specific effects [6].

SHARPIN is a highly conserved protein that is widely expressed in cells and tissues of mammalian species. It contains three domains, an amino-terminal coiled-coil (CC) domain, an ubiquitin-like (UBL) domain, and an Npl4 zinc finger (NZF) domain [7–9]. SHARPIN was originally described as a structural component of the postsynaptic density of the excitatory synapse in the rat brain where it interacts with SHANK proteins via its CC domain [10]. More recently, SHARPIN was identified as a component of the linear ubiquitin chain assembly complex (LUBAC) which is required for activation of the NFkB signaling pathway [11–14]. LUBAC is composed of an E3 ubiquitin ligase, RING finger protein 31 (RNF31, also known as HOIP), associated with RanBP-type and C3HC4-type zinc finger-containing protein 1 (RBCK1, also known as HOIL1) and SHARPIN. TNF induced cell death is a driver of the cell death seen in the skin of *Sharpin*^*cpdm*^ mice as evidenced by the sensitivity of SHARPIN-deficient cells to TNF-induced necroptosis and the lack of dermatitis in TNF-deficient *Sharpin*^*cpdm*^ mice [15, 16] TNFR1 results in recruitment of LUBAC to a signaling complex that initiates NFkB signaling. LUBAC catalyzes the addition of linear ubiquitin chains, connected by Met1 linkages, to RIPK1 and NEMO. The degree of ubiquitination is carefully controlled by deubiquitinases, in particular the ubiquitin carboxyl-terminal hydrolase CYLD that hydrolyzes Lys63 and Met1 linkages, and OTULIN that is specific for linear ubiquitin chains [17–19]. Preliminary observations of compound mutant mice (*Sharpin*^*cpdm*^ and *Cyld*^*tm1Scs*^) suggest that the *Sharpin*^*cpdm*^ phenotype may be due to the failure to inactivate CYLD (https://www.biorxiv.org/content/10.1101/2020.01.27.919076v1.full). Genetically determined deficiencies in RNF31 and RBCK1 cause immune deficiency, autoinflammatory disease, and glycogen storage disease in human patients [20–23], whereas absence of OTULIN has been reported in six patients with an autoinflammatory syndrome [24, 25].

Fibroblasts, B cells, and dendritic cells isolated from patients or mice deficient in LUBAC components have decreased NFkB signaling in response to TNF and TLR agonists resulting in decreased activation and secretion of cytokines. These findings seem inconsistent with the inflammatory phenotype of LUBAC-deficient patients and mice. Similarly, the association of autoinflammatory disease with both increased and decreased linear ubiquitination and the coexistence of immunodeficiency and autoinflammation in RNF31- and RBCK1-deficient patients seem contradictory. Fibroblasts from patients multi-organ autoinflammation, immunodeficiency, and amylopectinosis due to loss of function mutations in RBCK1 and RNF31 exhibited diminished IL1B- and TNF-induced NFκB activation yet monocytes were hyper-responsive to IL1B [26]. These observations point to tissue and cell-specific roles of factors associated with linear ubiquitination. This is also suggested by the reduced response to IL1B in fibroblasts and increased response in whole blood or monocyte cultures from patients with RBCK1- and RNF31-deficiency [21]. In *Sharpin*^*cpdm*^ mice IL1B modulates the cutaneous inflammation [27]. In addition to the role of SHARPIN as a structural component of the excitatory synapse and of LUBAC, SHARPIN suppresses B1-integrin activation [28, 29]. There are many other molecular pathways affected directly and indirectly [15, 16, 30–34].

To date, absence of SHARPIN has not been reported in human patients. However, autosomal defects in LUBAC, which SHARPIN is part of, are associated with autoinflammatory and immunodeficiency diseases in humans [35]. Increased expression of SHARPIN was reported in different types of cancers in human patients and appears to be associated with increased malignant behavior [36–41]. This may be in part due to its role in increasing neovascularization in cancer models [42].

The mechanism of carbon tetrachloride and acetaminophen-induced hepatic cirrhosis in mice was shown to be the effects of reduction of SHARPIN in the liver of treated mice [43] and it may play a role in nonalcoholic steatohepatitis [44].

The complex phenotype affecting multiple organ systems evidenced in *Sharpin* mutant mice suggests that *Sharpin* function may vary according to cell type. Many of the studies that detail the biochemical studies of SHARPIN function cited above were done *in vitro* using immortalized mouse embryonic fibroblast cell cultures. In primary cell cultures SHARPIN deficiency sensitized mouse and human keratinocytes as well as mouse embryonic fibroblasts to TNF-induced apoptosis [15]. We hypothesized that the variation in results was because the mouse *Sharpin* gene is primarily expressed in keratinocytes not fibroblasts. To investigate this, a conditional *Sharpin* null (B6(Cg)-*Tyr*^*c-2J*^ *Sharpin*^*tm1Sun*^/Sun) was created that developed large differences in disease severity, anatomic involvement, to normality depending upon which *cre*-recombinase was used indicating anatomic site specificity.

## Methods and materials

### Source and management of mice

All mice were maintained in the humidity, temperature, and light cycle (12:12) controlled vivarium under specific pathogen-free conditions (http://jaxmice.jax.org/genetichealth/health_program.html). Mice were housed in double-pen polycarbonate cages (330 cm^2^ floor area) at a maximum capacity of four mice per pen. Mice were allowed free access to autoclaved food (NIH 31, 6% fat; LabDiet 5K52, Purina Mills, St. Louis, MO) and acidified water (pH 2.8–3.2). All work was done with the approval of The Jackson Laboratory Animal Care and Use Committee under approval number 07005.

All mice used in these studies were obtained from or created at The Jackson Laboratory (Bar Harbor, ME). The *neo* cassette was removed by reciprocal matings between homozygous B6(Cg)-*Tyr*^*c-2J*^ (stock no. 000058), B6(Cg)-*Sharpin*^*tm1Sun*^/Sun to create an albino strain. Mice negative for the *neo* cassette were bred back to the strain of origin, B6(Cg)-*Tyr*^*c-2J*^/J to remove the *flp* allele and mice heterozygous for *Sharpin* and *flp* negative were mated to produce a homozygous colony. B6. Cg-Tg(ACTFLPe)9205Dym/J (stock no. 005703), both homozygous female and male mice, were mated to homozygous female and male B6(Cg)-*Tyr*^*c-2J*^ *Sharpin*^*tm1*.*1Sun*^/Sun mice to remove the *neo* cassette (stock no. 012631). The resulting mice were crossed with BALB/c-Tg(*CMV-cre*)1Cgn/J (stock no. 003465) or B6.C-Tg(*CMV-cre*)1Cgn/J (stock no. 006054) to remove *Sharpin* expression in all cells (ubiquitous expression). In order to only remove *Sharpin* expression from keratinocytes, B6(Cg)-*Tyr*^*c-2J*^ *Sharpin*^*tm1Sun*^/Sun with the *neo* removed were crossed with STOCK Tg(*KRT14-cre*)1Amc/J (stock no. 004782). To eliminate *Sharpin* expression from adipocytes, hemizygous male B6.FVB-Tg(*Adipoq-cre*)1Evdr/J (stock no. 010803) mice were mated to female B6(Cg)-*Tyr*^*c-2J*^ *Sharpin*^*tm1Sun*^/Sun. To remove *Sharpin* from fibroblasts the mice were crossed with BALB/c-Tg(*S100a4-cre*)1Eng/YunkJ (stock no. 012641). F1 mice, heterozygous for both *Sharpin* and *cre*-recombinase, were crossed to produce F2s that were genotyped for *Sharpin* and for *cre*-recombinase using the generic *cre* quantitative QPCR using protocols described on The Jackson Laboratory’s website (http://jaxmice.jax.org) for the respective strain.

### Creation of *Sharpin* conditional null mice

**Fig 1:**
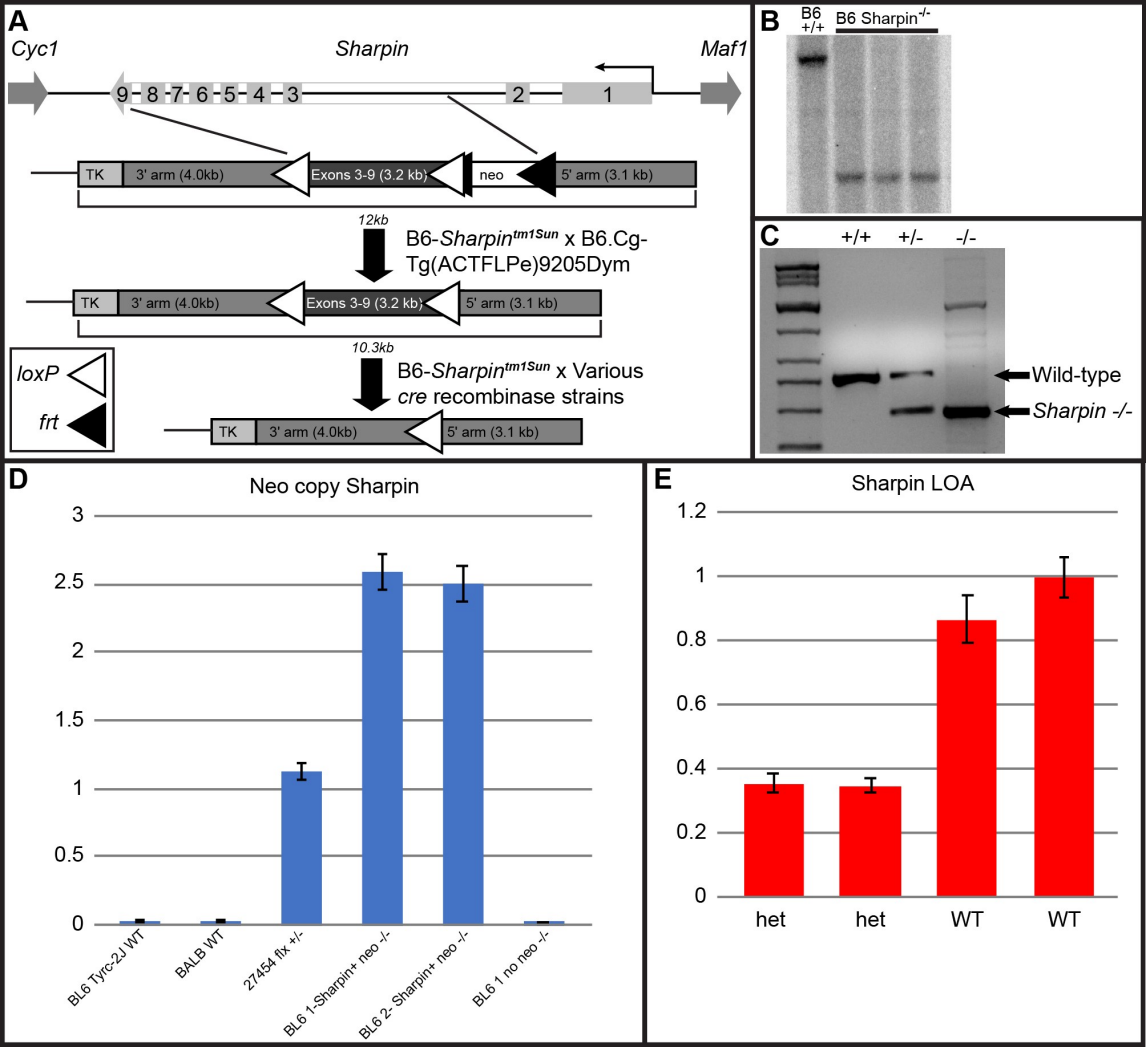
Identification of correctly targeted ES cell clones. Homologous recombination between the *Sharpin* cko-targeting vector and the *Sharpin* genomic locus, flanked by *Cyc1* and *Maf1*. The correctly targeted locus is 12kb, when mated to *Flpe*, the removal of neo will leave a 10.3 kb targeted allele and mating to various *cre-*recombinase strains will leave 7kb for the *Sharpin*^-/-^ allele. LoxP sites are depicted by white triangles and *frt* sites by black triangles (A). Southern blot analysis of the ES cell clones. (B6+/+) Wild-type ES clones; (B6 *Sharpin*) conditional knockout ES clones. Correctly targeted ES cells (cko allele) have a 5.0-kb DraIII band, the wild type allele shows 8.5 kb band, following hybridization with the 5’ probe (B). Genotyping assay for *Sharpin* alleles. Primers were designed to discriminate between the *Sharpin* alleles, WT+/+, het +/-, and null -/-. Arrows point to the expected size band, wild-type or *Sharpin*^-/-^. Lane 1 shows molecular weight marker (C). Neo copy Sharpin. A copy number QPCR assay was performed on the various *Sharpin* alleles. Lane 1 and 2- BL6 *Tyr*^*c-2J*^ and BALB WT do not contain any neo copies, lane 3–27454 *Sharpin* het (+/-) post *cre*, pre *Flpe* shows a single neo copy, lanes 4 and 5 are homozygous null pre *Flpe*, and lane 6 is BL6-1 after *Flpe* mating (D). *Sharpin* loss of allele (LOA). QPCR LOA assay was performed to detect the presence of absence of the *Sharpin* exon 4. The first 2 het samples show the loss of a *Sharpin* allele as compared with the WT sample (E).

To create a conditional knockout line, a *Sharpin* conditional targeting vector was generated using standard recombineering techniques [45]. A mini-retrieval vector was prepared using PCR products of 500 bp for the 5’ and 3’ mini homology segments (*Sharpin* 5’ret-F 5’-GCGGCCGCAACTCAGCACTGGCTGACAG-3’ and *Sharpin* 5’ret-R 5’GGATCC GCTGCAGTGCCTCATGGGAC-3’ and Sharpin 3’ret-F 5’-GGATCCAGGCGACCCAGCATAGAA-3’, *Sharpin* 3’ret-R 5’-CTCGAGTGAGCTCAAGGTTCATGCATG-3’) which contained restriction sites for cloning into *pBlight* (a plasmid with a MCI_TK cassette downstream of the 3’ retrieval arm, gift of S. Warming [45]). The linearized mini-retrieval vector was used to retrieve 9.6 kb of *Sharpin* and flanking genomic sequence from BAC RP23-204K8 (Children's Hospital Oakland Research Institute, Oakland, CA) by gap repair. The retrieved genomic sequence contained the entire *Sharpin* gene of 4.07 kb including 9 exons of which 8 are coding, as well as 978 bp of *Maf1* sequence 5’ of the *Sharpin* gene, and the 2.1 kb gene sequence for *Cyc1* present within the region comprising the 4kb 3’ homology arm. The placement of the *frt-PGK-neo-frt* cassette was designed to avoid repetitive sequences in intron 2 of *Sharpin* using the online application RepeatMasker (http://www.repeatmasker.org/cgi-bin/WEBRepeatMasker). Exons 3–9 of the *Sharpin* gene (3kb) were floxed using *loxP* sites that were inserted by recombineering. On the 3’ end of exon 9 a floxed *neo* cassette was engineered that was subsequently exposed to *cre-*recombinase to leave a single *loxP* site on the 5’ end of *Sharpin* exons 3–9. In intron 2, a *frt-PGK-neo-frt loxP* cassette was inserted by recombineering, to effectively flank functionally essential parts of the gene with *loxP* sites for removal in a temporal and spatial manner by breeding to a *cre*-expressing strain. The *PGK*-*neo* cassette was flanked by *frt* sequences to facilitate its removal independent of the *loxP*. The *frt-PGK-neo-frt-loxP* cassette was preceded by a 2.3 kB arm of the *Sharpin* gene sequence. 3’ of the floxed *Sharpin* exons is 4kb of sequence comprising the 3’ retrieval arm (Fig 1A).

The correct sequence was confirmed by Sanger sequencing. The plasmid was purified using the Midi-prep kit (Qiagen, Valencia, CA).

Plasmid DNA (60 ug) was linearized with *Sspl* precipitated with ethanol and submitted to the Cell Biology Core at The Jackson Laboratory for B6-*Tyr* embryonic stem (ES) cell electroporation. Stably transfected cells were selected by *G418* resistance. A total of 360 B6-*Tyr* ES cell clones were screened for homologous insertion of the transgene by Southern blot after digestion with *DraIII*, using as a probe a PCR product (primers Forward 5'- AGCTTACCTGCTGGGACTGAGG-3′ and Reverse 5'- GGAGCTAGGTAGCCATGCTGG -3') whose sequence lay outside of the 5’ homology arm.

Primers used for the detection of *Sharpin* post *flp* and post *cre* were GGGATGTATCTGTCAGGGAAC-mutant, GCCCTTGGAGGCTATTTGTT -common, and GCCCAGCTTTTCCATCACTA -wildtype reverse.

### Phenotyping mutant mice

For each comparison conducted in this study, age matched female and male mutant and control mice were collected, euthanized by CO_2_ asphyxiation using approved methods, and complete necropsies performed using previously described methods [46]. Briefly, hematoxylin and eosin (H&E) stained slides were examined by experienced board certified veterinary anatomic pathologists (JPS, HH) and all lesions subjectively scored (normal, 0; mild, 1; moderate, 2; severe, 3; extreme, 4) and data (with diagnosis and anatomic site) entered into the Mouse Disease Information Database (MoDIS) [47]. These data were used to generate spreadsheets for semi-quantitative analysis. Morphometric analyses of tissue sections were conducted to determine epidermal thickness (dorsal interscapular skin) along the linear length of sample. In each case, five measurements were made along an H&E stained section of dorsal skin from each mouse. The thickness of the Malpighian layer (basement membrane to the base of the stratum corneum) was measured in vertical sections (perpendicular to the basement membrane) in which the entire length of the hair follicle was visible in the field to assure consistent orientation. Stratum corneum was measured immediately above the Malpighian layer. The thicknesses of both layers were added to yield the total epidermal thickness of the sample. Measurements were done manually at 400x magnification using a DP27 digital camera on a BX50F4 photomicroscope (Olympus, Tokyo, Japan) and DP controller 3.2 software (Olympus, Center Valley, PA) by one pathologist (JPS).

### Hematology

Whole blood (200 μl) was collected in soda lime glass micro-hematocrit capillary tubes (stock no. 51608, Globe Scientific, Paramus, NJ). The uncoagulated blood was run without separation on a Siemens Advia 2120 Hematology Analyzer (Siemens Healthcare Diagnostics Inc., Tarrytown, NY).

### Immunohistochemistry

To determine where SHARPIN protein was expressed, immunohistochemistry was done using a Ventana XT autostainer (Tuscon, AZ) with an antibody directed against SHARPIN (Sharpin (Y-14) stock no. sc~98129, Santa Cruz, Dallas, TX). Immunohistochemistry was tested using 5 different fixatives. Only 4% paraformaldehyde fixed sections worked with this antibody. For immunohistochemistry, 1 male 121 day old C57BLKaLawRij-*Sharpin*^*cpdm*^/RijSunJ (null mutant), 1 male 133 day old C57BLKaLawRij/RijSunJ (wildtype control), one 57 day old CByJ.OcB3-*Sharpin*^*cpdm-Dem*^/Sz (null mutant), and 1 CBy.OcB3/Sz (wildtype control) had skin collected and fixed in 4% paraformaldehyde. Slides were run using a Ventana Discovery XT autostainer (Ventana, Tuscon, AZ) with diaminobenzidine (Sigma, St. Louis, MO) used as chromogen.

### Quantitative real-time RT-PCR (qRT-PCR) to genotype for *Sharpin*

The expression of mRNA in the skin of 4-week old mice was determined by qRT-PCR [48]. Skin from 4 weeks of age/sex matched mice was collected and stored in RNALater (Qiagen, Valencia, CA) at -80°C until samples from all replicates were collected. RNA was then extracted using a PureLink RNA Mini Kit (Invitrogen, Grand Island, NY). For each qRT-PCR, a 15ul reaction was run with 7.5 μl Taqman One-Step RT-PCR Master Mix 2X (Life Technologies, Grand Island, NY), 0.4ul, 40X Multiscribe and Rnase Inhibitor Mix, 0.75 μl of 20X Assays on Demand Taqman primer and probe set and 100ng RNA. The qRT-PCR was performed in a Mastercycler® realplex4 (Eppendorf, Hauppauge, NY) programmed at 40 cycles of 42 C for 50 minutes, 90 C for 10 minutes, 95 C for 15 seconds, 60 C for 1 minute, and 72 C for 1 minute. The Ct values for each chemokine were normalized by subtracting the Ct values for the housekeeping gene *Actb* (Delta Ct). The relative fold-change in mRNA expression between wild-type mice and mutant mice was calculated by the 2^-(delta-delta Ct)^ method [49].

### Localization of gene expression for *cre*-recombinase transgenic mice

While a review of the literature can suggest where genes are naturally expressed, it is always important to verify this when various promoters are used with *cre*-recombinases. To do this, slides were obtained from the Comprehensive *cre* Characterization Resource at The Jackson Laboratory where whole slide scanned images of embryos (E15.5) or tissues (at P7 and P56) can be viewed online (http://www.informatics.jax.org/home/recombinase) [50]. The slides were created using frozen sections of tissues (or embryos) from crosses between each of the *cre*-recombinase transgenic mice listed above and B6.129S4-*Gt (Rosa) 26Sor*^*tm1Sor*^/J (Stock No. 3474). Fresh frozen sections were labeled as previously described [51].

### Serum immunoglobulins and serum IL-18

Concentrations of serum IgM, IgG, IgA, and IgE were determined by ELISA using isotype-specific reagents obtained from Southern Biotech (Birmingham, AL) as previously described [3]). The concentration of IL18 in serum of mice was determined using a commercially available ELISA (MBL International, Woburn, MA).

### Cytokines

RNA isolation and analysis from skin samples was done as previously described [52]. Skin samples collected from the left side of mice were homogenized in TRI-reagent (Sigma-Aldrich). Total RNA was isolated by standard TRI-reagent methods according to the manufacturer’s protocols and quantitated at 260 nm. RNA was analyzed by quantitative real time RT-PCR (qPCR) and custom eTags^™^ multiplex assays (ACLARA Biosciences, Mountain View, CA).

For qPCR, RNA was reverse transcribed for 1 hour at 42°C in 30μL volumes containing 0.5μg RNA, 2.8U Recombinant RNasin Ribonuclease Inhibitor, 400U M-MLV Reverse Transcriptase, 6.0μL M-MLV Reverse Transcriptase 5X buffer, 0.25μg Oligo(dT) 15 Primer, and 0.5mM of each nucleotide (Promega, Madison, WI) in a Peltier Thermal Cycler 200 (MJ Research/Bio-Rad Laboratories, Waltham, MA). The cDNA was analyzed with *Assays on Demand* Taqman® primer and probe sets following the manufacturer’s instructions using the 7300 Real Time PCR System and Sequence Detection Software v1.2.1 (Applied Biosystems, Foster City, CA). The Ct values for each chemokine were normalized by subtracting the Ct values for the housekeeping gene actin beta (*Actb*, ID Mm00607939_s1). The difference in mRNA expression between mice was calculated with the difference in normalized Ct values (fold change = 2^−ΔΔCt^) [49].

### Statistical analysis

Graphs and descriptive statistics were generated in GraphPad Prism Version 8.3.1. The statistical significance of differences between means of experimental groups for epidermal thickness, expression of mRNA and protein concentrations was determined by two-tailed Student’s t-test using Microsoft® Excel and GraphPad Prism. Differences were considered significant at p < 0.05.

## Results

### Creation of the conditional *Sharpin* allelic mutation

The wild-type *Sharpin* allele consists of 9 exons, which encode 387 amino acids, producing a 40kd protein (Fig 1). An alternative start site is hypothesized to exist at exon 6 that encodes a 60 kd splice variant (www.ensembl.org). Given the high number of repetitive sequences in intron 2, a targeting vector was constructed in which exons 3–9 were flanked by *Frt*-*neo-Frt-loxP* and *loxp* sites.

Mice positively expressing the new allele that also contained the *Frt-neo-Frt*-cassette had the latter cassette removed by crossing the mice with by reciprocal matings between homozygous B6(Cg)-*Tyr*^*c-2J*^ and B6(Cg)-*Sharpin*^*tm1Sun*^/Sun, to create an albino stock, and B6.Cg-Tg(ACTFLPe)9205Dym/J mice (Fig 1). Mice negative for the neo cassette were bred back to the strain of origin, B6(Cg)-*Tyr*^*c-2J*^/J to remove the *flp* allele and mice heterozygous for *Sharpin* and *flp* negative were mated to produce a homozygous colony. Once viable conditional *Sharpin* null (B6(Cg)-*Tyr*^*c-2J*^ *Sharpin*^*tm1*.*1Sun*^/Sun, hereafter referred to as *Sharpin*^-/-^) mice were confirmed, crosses were established with transgenic mice carrying cell specific promoters driving *cre*-recombinase in order to ubiquitously or selectively inactivate *Sharpin*.

### Expression of SHARPIN protein in various organs

**Fig 2:**
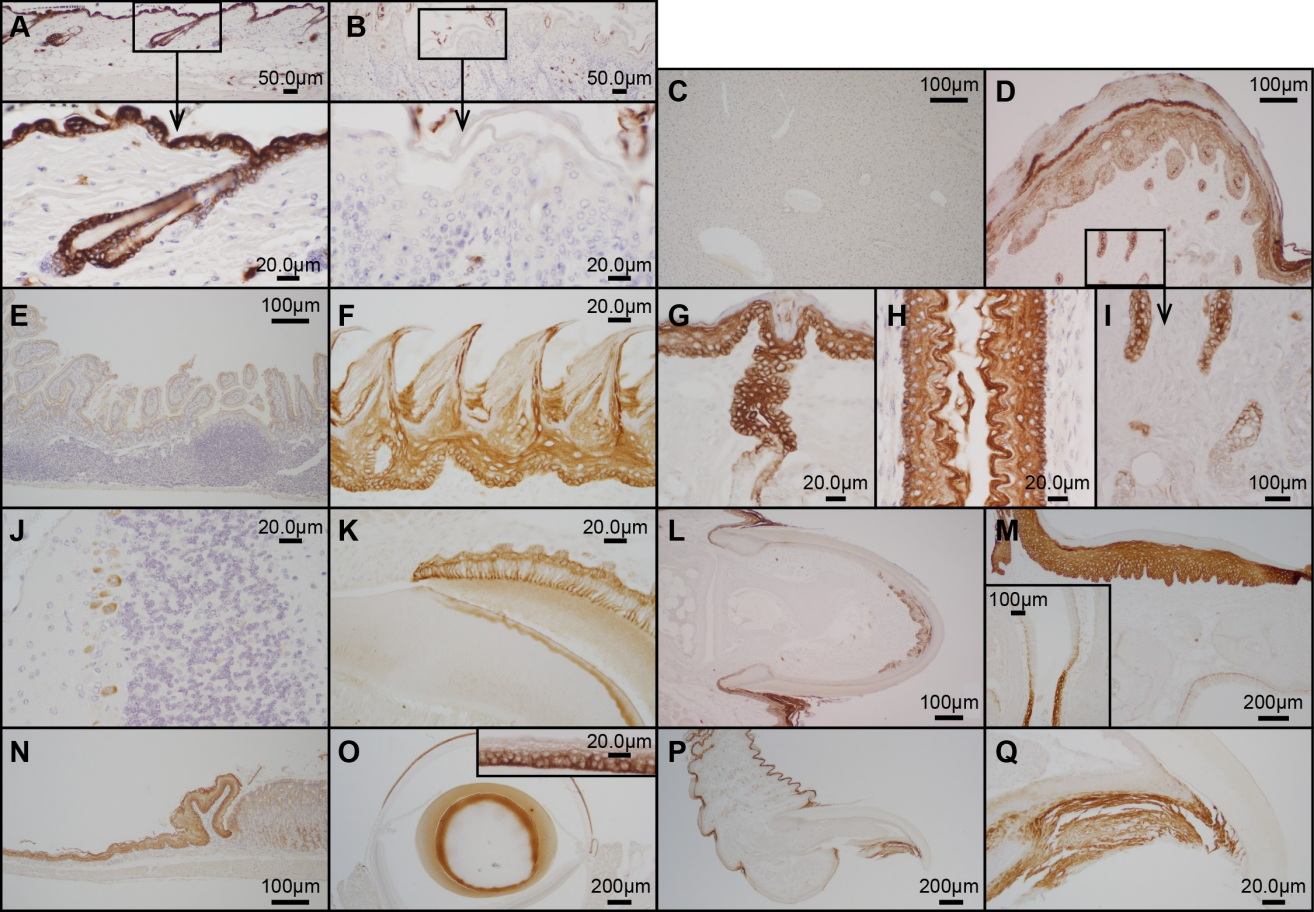
Immunohistochemical localization of SHARPIN protein. SHARPIN protein was normally expressed in the epidermis and hair follicles (A) of wild type, control mice (N = 2) but not in the spontaneous chronic proliferative dermatitis (*Sharpin*^*cpdm/cpdm*^) null mutant mice (N = 2, B), which served as a negative control. In normal wild type mice SHARPIN was not expressed in the liver (C), Peyer’s patches, or intestine (E). However, SHARPIN was expressed in epithelial cells of the foot pad (D) and eccrine gland (D, I), tongue (F), lingual gland (G), esophagus (H), hard palate (M) and nasal epithelium (M and insert), tooth (ameloblasts) (K), nail unit (hyponychium and proximal nail fold, L, P, Q), forestomach (N), cornea and lens of the eye (O), and Purkinje cells of the cerebellum (J).

To determine the distribution of SHARPIN protein expression, immunohistochemistry was done on tissues from wild type controls and *Sharpin*^*cpdm*^ mutant mice. Tissues from the *Sharpin*^*cpdm*^ null mice were negative (except for endogenous peroxidase activity in a few cells such as mast cells) which served as a negative control. SHARPIN was not expressed in the liver, Peyer’s patches, or intestine. However, SHARPIN was detected in epithelial cells of the skin and hair follicles, foot pad, eccrine gland, tongue, lingual gland, esophagus, hard palate, nasal epithelium, tooth (ameloblasts), nail unit (hyponychium and proximal nail fold), forestomach, cornea and lens of the eye, and Purkinje cells of the cerebellum (Fig 2).

### Expression of *cre*-recombinases in the skin

To verify that the transgenic mice carrying *cre*-recombinases expressed the *cre* where it was expected, they were crossed with transgenic mice carrying a LacZ reporter. The CMV ubiquitously expressing *cre*-recombinase exhibited widespread expression on the BALB/c and C57BL/6 background (data not shown). *Krt14-cre* transgene was expressed in the epidermis and hair follicles (and other epidermal structures) similar to immunohistochemical localization of KRT14 (S1A Fig) [53]. S100 calcium binding protein A4 (S100A4) is still published as fibroblast-specific protein [54], but the *S100-cre* transgene was detected in the epidermis and infundibulum of hair follicles (S1B Fig). *S100a4-cre* transgenic mice exhibited less expression in basal keratinocytes and the base of hair follicles relative to the *Krt14-cre* mice which probably accounts for the reduced severity when crossed with the inducible *Sharpin* null mice. The *Adipoq-cre* transgene was expressed in adipose tissue (S1C Fig). Additional images are available online (http://www.informatics.jax.org/home/recombinase) [50].

### Overall comparisons of phenotypes

Control mice and all crosses survived until at least 10 weeks of age with exception of the B6-*CMV-cre Sharpin*^-/-^ mice (hereafter all compound mutants will have *cre* left out), most of which died by 6 weeks of age. Therefore, most of the comparative studies were done at 6 weeks of age. BALB-*CMV Sharpin*^-/-^, B6-*CMV Sharpin*^-/-^, and *Krt14 Sharpin*^-/-^ mutant mice developed extensive dermatitis similar to the two spontaneous allelic null mutations, *Sharpin*^*cpdm/cpdm*^ and *Sharpin*^*cpdm-Dem/cpdm-Dem*^ with no sexual dimorphism [1, 2]. The mice had severe pruritus and scratched frequently which led to skin erosions and ulcers necessitating euthanasia at 8–10 weeks of age for the BALB-*CMV Sharpin*^-/-^ and *Krt14 Sharpin*^-/-^ mice.

Body weights were measured on at least 5 mice of each genotype, females and males. Males were consistently higher in weight than females. Control mice and mutant mice with no or mild skin lesions gained weight during the 4–10 week of age observation period (S2 Fig). Degree of failure to gain weight reflected the severity of systemic disease in each cross due to loss of *Sharpin*. The ubiquitously expressing *cre*-recombinases resulted in the most severe disease and failure to survive or gain weight. Those with primarily skin lesions (*Krt14* cross) were moderately affected. Those with no (*Adipoq*) or relatively minor lesions, primarly referring to the skin disease (*S100a4*) were within or at the bottom of the normal range for gain of body weight. The B6-*CMV Sharpin*^-/-^ mice gained little weight until they died by 6 weeks of age. The BALB-*CMV Sharpin*^-/-^ mice gained some weight but lagged below the other crosses. The *Krt14 Sharpin*^-/-^ mice had moderate weight gain but less than the control mice whereas *S100a4 Sharpin*^-/-^ and *Adipoq Sharpin*^-/-^ mice were within the normal range for body weight.

Mice carrying the spontaneous *Sharpin*^*cpdm/cpdm*^ mutation had marked changes in the peripheral blood white blood cell count and differentials [6]. Changes in the blood confirmed the chronic proliferative dermatitis phenotype in the ubiquitously expressing *CMV Sharpin*^-/-^ mutant mice on both backgrounds but with a high degree of variability between individuals. However, there was a significant increase of neutrophils in both the *Krt14* and *S100a4 Sharpin*^-/-^ mice, but only eosinophils for the *Krt14 Sharpin* mutant mice (S3 Fig). As with the spontaneous *Sharpin* null mice [6], there was no sexual dichotomy. There was no change in the peripheral blood for the *Adipoq Sharpin*^-/-^ mutant mice.

**Fig 3:**
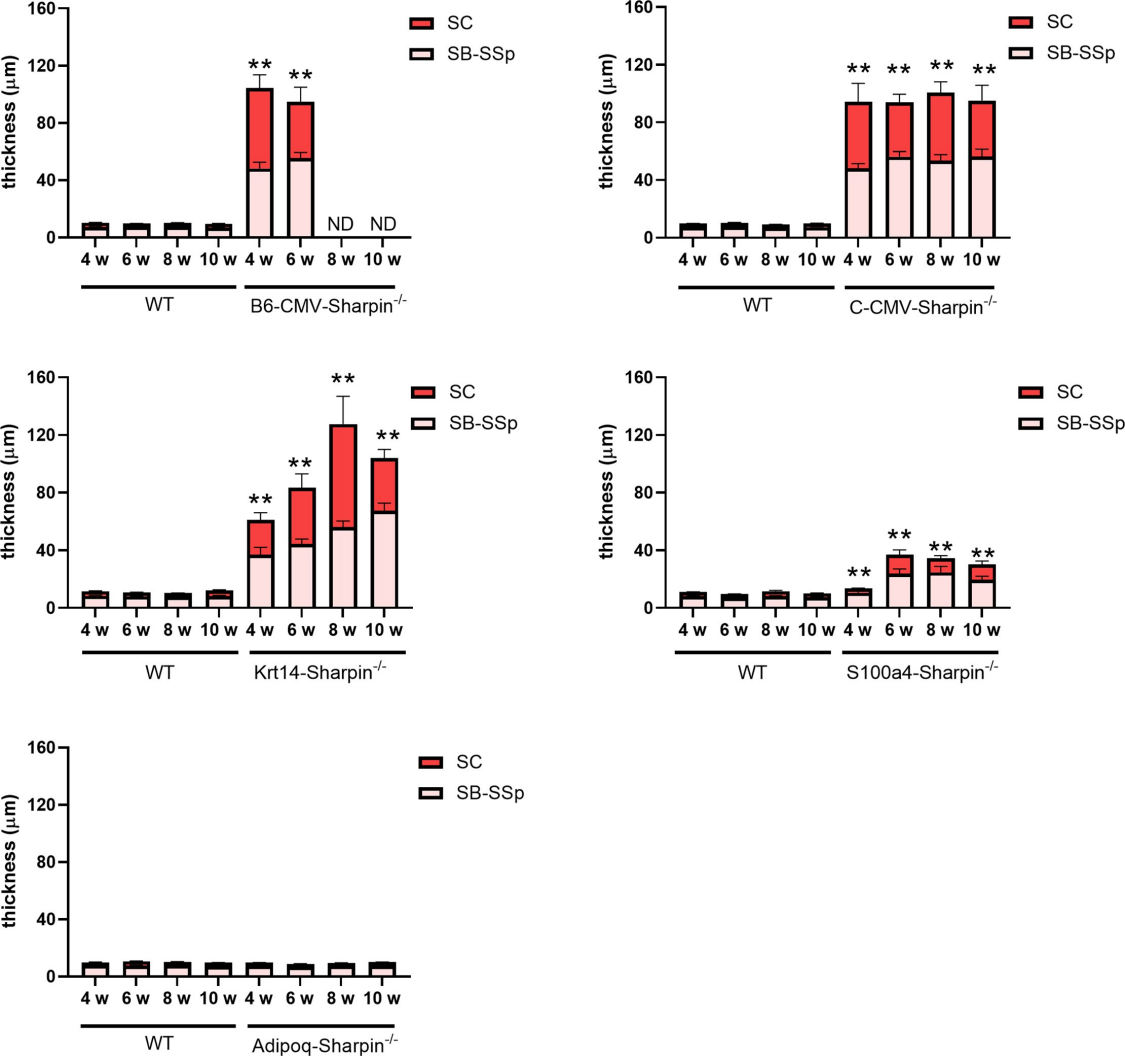
Changes in epidermal thickness. There was a significant difference in thickness of the stratum basale and stratum spinosum (SB-SSp) and the stratum corneum (SC) between the control mice (WT) and B6-*CMV*, C-*CMV*, *Krt14*, and *S100a4 Sharpin*^*-/-*^ mutant mice (** p < 0.01). Epidermal thickness was similar in the *Krt14 Sharpin*^*-/-*^ to those ubiquitously expressing CMV-*cre*, but less in *S100a4 Sharpin*^*-/-*^ mice. The *Adipoq Sharpin*^*-/-*^ mutant mice were unaffected, identical to the controls. Bars represent the mean + SEM of 4–10 mice per group. ND–not done.

Epidermal hyperplasia is a hallmark of the chronic proliferative dermatitis phenotype [1, 6] and a useful marker for quantitative assessment of the dermatitis. The thickness of both the Malpighian layer (stratum basale and stratum spinosum) and the stratum corneum in both the B6-*CMV Sharpin*^-/-^ and BALB-*CMV Sharpin*^-/-^ mutant mice was significantly greater than the controls, similar to that found the two spontaneous allelic null mutations (*Sharpin*^*cpdm/cpdm*^ and *Sharpin*^*cpdm-Dem/cpdm-Dem*^) (Fig 3) [6]. *Krt14 Sharpin*^-/-^ mice had a similar increase of epidermal thickness as mice with ubiquitously deleted *Sharpin*. The *S100a4 Sharpin*^-/-^ mice had a thicker epidermis than controls but it was significantly less so than the *CMV Sharpin*^-/-^ and *Krt14 Sharpin*^-/-^ crosses. There was no difference in epidermal thickness between the *Adipoq Sharpin*^-/-^ and control mice (Fig 3).

### *Cre*-specific phenotypes

Lesions varied by their presence or absence in different organs as well as the severity in specific organs based on the *cre*-recombinase used. Lesions were compared with those in the spontaneous null allele *Sharpin*^*cpdm/cpdm*^ (Table 1). Major lesions are described by each cross and the accompanying figures compare lesions from the same sites for all the crosses.

**Table 1:** Histologic lesions in wild type and mutant 6 week old mice. Note variation in lesion distribution and severity based on where SHARPIN was deleted.

Strain	Skin	Forestomach	Liver	Lung	Shoulder	Knee	Middle Ear	Peyer’s Patch	Spleen	Lymph Node
*Sharpin* ^*+/-*^	**Normal**	**Normal**	**Normal**	**Normal**	**Normal**	**Normal**	**Normal**	**Normal**	**Normal**	**Normal**
*Sharpin*^*cpdm/cpdm*^	**Severe**	**Severe**	**Severe**	**Severe**	**Severe**	**Severe**	**Severe**	**Missing**	**Severe**	**Severe**
BALB-*CMV-cre Sharpin*^*-/-*^	**Severe**	**Severe**	**Severe**	**Mild**	**Variable**	**Minimal**	**Severe**	**Missing**	**Severe**	**Severe**
B6-*CMV-cre Sharpin*^*-/-*^	**Severe**	**Severe**	**Severe**	**Severe**	**Normal**	**Mild**	**Severe**	**Missing**	**Severe**	**Severe**
*Krt14-cre Sharpin*^*-/-*^	**Severe**	**Normal**	**Normal**	**Normal**	**Normal**	**Normal**	**Normal**	**Normal**	**Normal**	**Normal**
*S100a4-cre Sharpin*^*-/-*^	**Mild**	**Normal**	**Normal**	**Severe**	**Severe**	**Mild**	**Normal**	**Normal**	**Normal**	**Mild**
*Adipoq-cre Sharpin*^*-/-*^	**Normal**	**Normal**	**Normal**	**Normal**	**Normal**	**Normal**	**Normal**	**Normal**	**Normal**	**Normal**

F1 mice heterozygous for both *Sharpin* and *cre*-recombinase were crossed to produce F2 mice that were genotyped for *Sharpin* and for *cre* using quantitative QPCR. All genotypes were evaluated but only those homozygous for *Sharpin*^*tm1*.*1Sun*^ and homozygous or heterozygous for the *cre*-recombinase developed lesions. Results were similar in mice carrying one or both copies of the *cre*-recombinase.

#### B6(Cg)-*Tyr*^*c-2J*^ *Sharpin*^tm1Sun^/Sun mice recapitulate the *cpdm* phenotype when crossed to mice ubiquitously expressing *cre*-recombinase

Conditional *Sharpin* null mice were crossed with a *CMV* ubiquitously expressing *cre*-recombinase on two different genetic backgrounds. The BALB/c transgenic line was initially available, which resulted in a hybrid stock, potentially complicating interpretation of the phenotype. When the B6 transgenic line became available this construct was also used.

**Fig 4:**
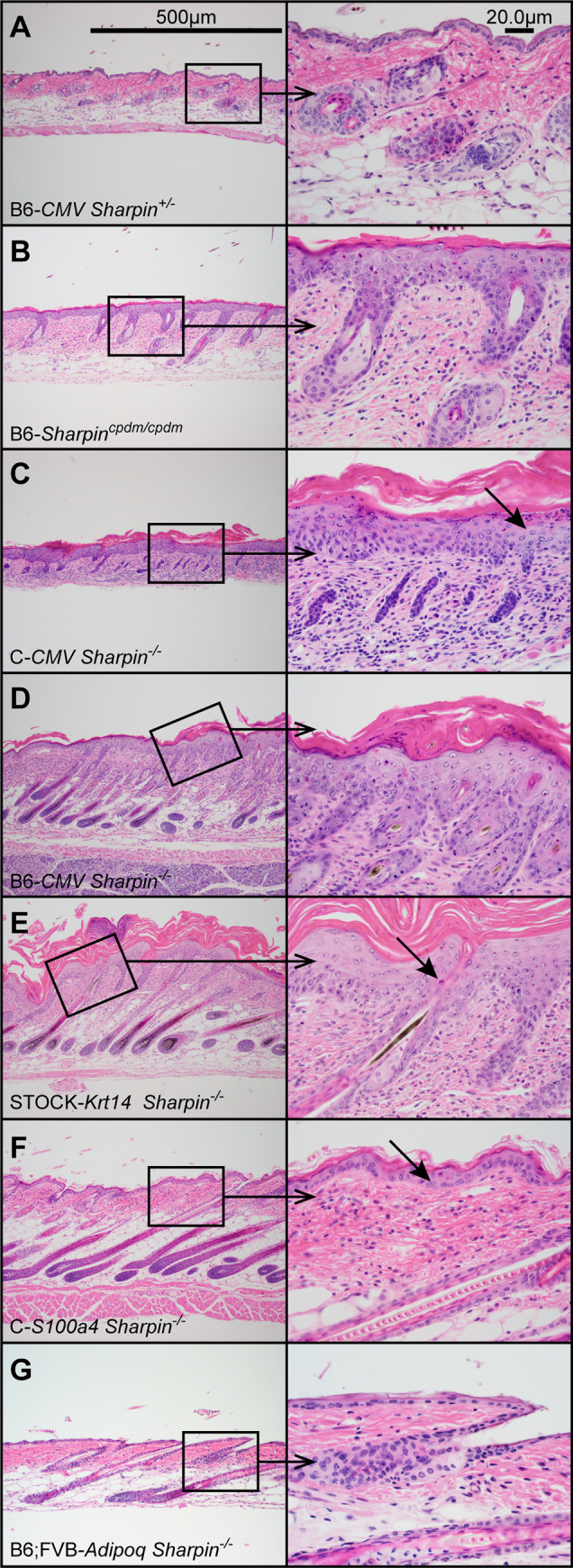
Dorsal skin histology. Female mice, 6 weeks of age, carrying any of the *cre*-recombinase transgenes but no or only one copy of the conditional *Sharpin* null gene were normal (A). Normal epidermis was thin with few, if any inflammatory cells in the dermis. However, mice carrying one or two copies of the ubiquitously expressing *CMV-cre* and homozygous for the conditional *Sharpin* gene (*Sharpin*^*-/-*^) (C, D) had lesions identical to the spontaneous *Sharpin*^*cpdm/cpdm*^ mice (B). The epidermis was moderately to severely acanthotic with orthokeratotic hyperkeratosis. Apoptosis (arrows) of keratinocytes was common. Dermis had various degrees of inflammation consisting of a mixture of eosinophils, neutrophils, and macrophages. Lesions were the same for the *Krt14* mice (E) but much less severe, although present, in the *S100a4* mice (F). *Adipoq Sharpin*^*-/-*^were normal. Low magnification 100X, high magnification 400X (G).

Lesions were largely similar to what has been earlier described in *Sharpin*^*cpdm/cpdm*^ and *Sharpin*^*cpdm-Dem/cpdm-Dem*^ mice with the spontaneous null mutation [1, 6, 55]. The mice had prominent skin lesions (Fig 4). The epidermis contained scattered apoptotic keratinocytes (arrow, Fig 4E) and was significantly increased in thickness as a result of acanthosis and ortho- and parakeratotic hyperkeratosis (Fig 3). There was inflammation of the dermis comprised of accumulations of granulocytes and macrophages. Eosinophils were also present in the epidermis and occasionally formed intracorneal and subcorneal pustules. There were increased numbers of cross-sections of small blood vessels in the dermis as found in the spontaneous mutants [56]. The dermis of older mice had an increase of collagen deposition (fibrosis).

There was moderate to severe hyperplasia of the epithelium of the esophagus with scattered apoptotic epithelial cells (data not shown). The cornified layer of the epithelium was thickened and there was accumulation of eosinophils in the subepithelial propria mucosae and the basal layer of the epithelium. Changes were similar to those described in *Sharpin*^*cpdm/cpdm*^ mice [57].

Changes in the forestomach varied from the presence of a few apoptotic epithelial cells to marked and diffuse thickening of the epithelium (S4 Fig). A few inflammatory cells, mostly eosinophils, were present in the subepithelial connective tissue. Mild hyperplasia and a few apoptotic epithelial cells were observed on the epithelium of the oral cavity. These changes resemble those found in the spontaneous mutant mice [57].

There was mild to extensive inflammation in the portal areas and surrounding the central veins of the liver (S5 Fig), and around the bronchioles and blood vasculature of the lungs (S6 Fig). The inflammation in the liver consisted mostly of a mixed population of granulocytes and macrophages with mild fibrosis. In the lungs, the majority of cells were lymphocytic with fewer granulocytes and macrophages. Inflammatory changes were observed in the joints of some mice (S7 and S8 Figs). The most severely affected joint was the humeroscapular (shoulder joint) with accumulation of granulocytes in the synovial membrane and surrounding tissues and fibrosis. Milder changes were seen in the knee joints. Similar inflammatory changes were observed in the middle ear (S9 Fig).

Similar to *Sharpin*^*cpdm/cpdm*^ mice, in both of the *CMV-cre Sharpin*^-/-^ strains there was a nearly complete loss of lymphoid tissues in the small and large intestine (S10 Fig). In younger mice, small aggregates of lymphocytes often mixed with granulocytes, mostly eosinophils (confirmed using eosinophilic major basic protein by immunohistochemistry, data not shown), could occasionally be found in the submucosa and lamina propria of the small and large intestine. These represent remnants of the Peyer’s patches and large intestinal follicles following a process of involution as previously described [5].

Well-defined lymphoid follicles were absent from the white pulp of the spleen and from lymphoid follicles. There was a mild to moderate increase of myelopoiesis in the red pulp (S11 Fig). There was marked lymphoid depletion with effacement of the lymph node by eosinophils in the spontaneous and ubiquitously deleted *Sharpin* null mice (S12 Fig). The thymus of *Sharpin*^*cpdm/cpdm*^ mice was often smaller in size, but maintained a well populated cortex which was clearly separated from the medulla. By contrast, mild to severe involution of the thymus was present in *CMV*-*cre* mice. In the mildly affected mice, the overall thymus was markedly reduced in size, but there was a clearly defined cortex that was densely populated with thymocytes. In more advanced lesions, there was a marked increase of apoptosis of thymocytes in both the cortex and medulla. In the most advanced lesions, a single remnant of the thymus could be identified consisting of a small sheet of epithelial cells with few lymphocytes. Cysts lined by ciliated epithelial cells were present in the thymic remnant of one mouse.

#### *Krt14-cre*-recombinase mice

No lesions were observed in the control mice. The epidermis of *Krt14 Sharpin*^*-/-*^ mice contained numerous apoptotic keratinocytes and had marked acanthosis and ortho- and parakeratotic hyperkeratosis resulting in a significant increase of epidermal thickness (Figs 3 and 4E). The increased epidermal thickness was similar to that seen in the spontaneous and ubiquitously deleted *Sharpin* mutant mice. There was abundant mixed cellular inflammation in the dermis. Granulocytes, both eosinophils and neutrophils, were common in the dermis and epidermis and frequently formed intracorneal or subcorneal microabscesses. There were occasional apoptotic epithelial cells in the oral mucosa with mild acanthosis and focal areas of parakeratotic hyperkeratosis. A few granulocytes were present in the subepithelial propria mucosae and in the epithelium. No changes were observed in the mucosa of the esophagus or forestomach (S4E Fig) in contrast to *Sharpin*^*cpdm/cpdm*^ mice with generalized deletion of the SHARPIN protein. Lymphoid tissues appeared normal including the presence of Peyer’s patches in the small intestine (S10E Fig) and well-defined lymphoid follicles in lymph nodes (S12E Fig) and spleen (S11E Fig). There was a mild increase of extracellular hematopoiesis, primarily myelopoiesis, in the red pulp of the spleen of a few mice (S11E Fig). No evidence of inflammation was present in the liver (S5E Fig), lungs (S6E Fig), joints (S7E and S8E Figs) or middle ears (S9E Fig) of *Krt14 Sharpin*^*-/-*^ mice at 4, 6, and 8 weeks of age. However, one 10-week-old female mouse had severe inflammation characterized by accumulation of granulocytes and macrophages with fibrosis around central veins and in portal areas. There were no lesions in the lungs or joints in this mouse.

#### *S100a4-cre*-recombinase mice

No lesions were observed in the control mice. The epidermis of *S100a4 Sharpin*^*-/-*^ mice contained apoptotic keratinocytes and had various degrees of acanthosis and orthokeratotic hyperkeratosis resulting in mild to moderate increase of epidermal thickness (Figs 3 and 4F) which were less severe than seen in the ubiquitously deleted *Sharpin* or *Krt14 Sharpin*^-/-^ mice. Mild to moderate mixed cellular inflammation was present in the dermis with occasional presence of inflammatory cells in the epidermis. By contrast to *Sharpin*^*cpdm/cpdm*^ or *CMV Sharpin*^-/-^ mice with generalized deletion of the SHARPIN protein, lymphoid tissues appeared normal including the presence of Peyer’s patches in the small intestine (S10F Fig) and well defined lymphoid follicles in lymph nodes (S12F Fig) and spleen (S11F Fig). There was a mild to moderate increase of extracellular hematopoiesis and primarily myelopoiesis in the red pulp of the spleen. The shoulder (humeroscapular) joints of 15 of the 16 mice in which this joint could be evaluated had severe arthritis characterized by accumulation of fibrin in the joint space and infiltration of the synovia and surrounding tissues by granulocytes and macrophages (S7F Fig). Fibrotic changes were occasionally observed. There was no light microscopic evidence of damage to the articular cartilage. Mild to moderate arthritis and tendinitis was present in the knee joints of 10 mice. There was also inflammation of the temporomandibular joints in five of 13 mice in which these tissues could be examined (data not shown). The inflammation varied from mild accumulation of granulocytes to the presence of fibrin and numerous granulocytes. No evidence of inflammation was present in the elbow, phalangeal, and intervertebral joints. There was inflammation characterized by accumulation of granulocytes admixed with macrophages in the connective tissue at the base of the heart of four mice, extending into the base of the aorta in two mice. There was mild mixed cellular perivascular and peribronchiolar inflammation in the lungs and inflammation in periportal triads of the liver in one 10-week-old *S100a4 Sharpin*^*-/-*^ mouse. The lungs (S6F Fig) and liver (S5F Fig) of the other *S100a4 Sharpin*^*-/-*^ mice had no inflammation.

#### *Adipoq-cre* recombinase mice

There were no differences noted between the control mice and *Adipoq Sharpin*^*-/-*^ mice (Fig 3G, S6–S12G Figs).

### Serum immunoglobulins and serum IL18

**Fig 5:**
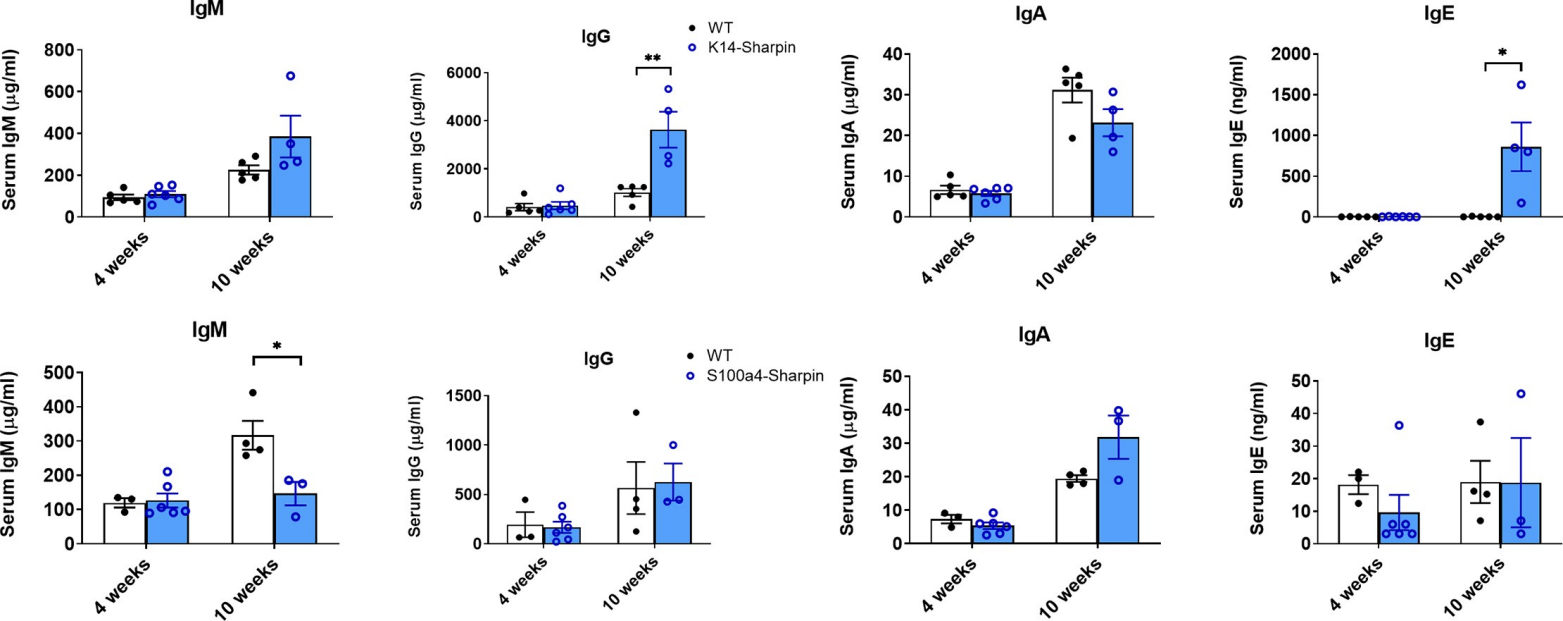
Serum immunoglobulins. Concentration of immunoglobulin in the serum of *Krt14 Sharpin*^-/-^ and WT mice (top panels) and *S100a4 Sharpin*^-/-^ and WT mice (bottom panels). Bars indicate the mean ± SEM of 3–6 mice per group. * p < 0.05; ** p < 0.01.

Mice with the spontaneous *Sharpin*^*cpdm/cpdm*^ mutation have a defect in isotype switching resulting in a marked decrease of total serum IgG and IgA, and nearly undetectable IgE (S13 Fig) [3]. *S100a4 Sharpin*^-/-^ mice had similar concentrations of IgG, IgA, and IgE as control mice, and a slightly reduced concentration of IgM (Fig 5). *Krt14 Sharpin*^-/-^ mice had normal immunoglobulin concentrations at 4 weeks of age, but significantly increased concentrations of serum IgG and IgE at 10 weeks of age (Fig 5). These data indicate that these mice with selective deletion of *Sharpin* in non-immune cells did not have a defect in isotype switching and, secondly, demonstrated that the dermatitis in the *Krt14 Sharpin*^-/-^ mice was associated with a marked increase of serum IgE.

**Fig 6:**
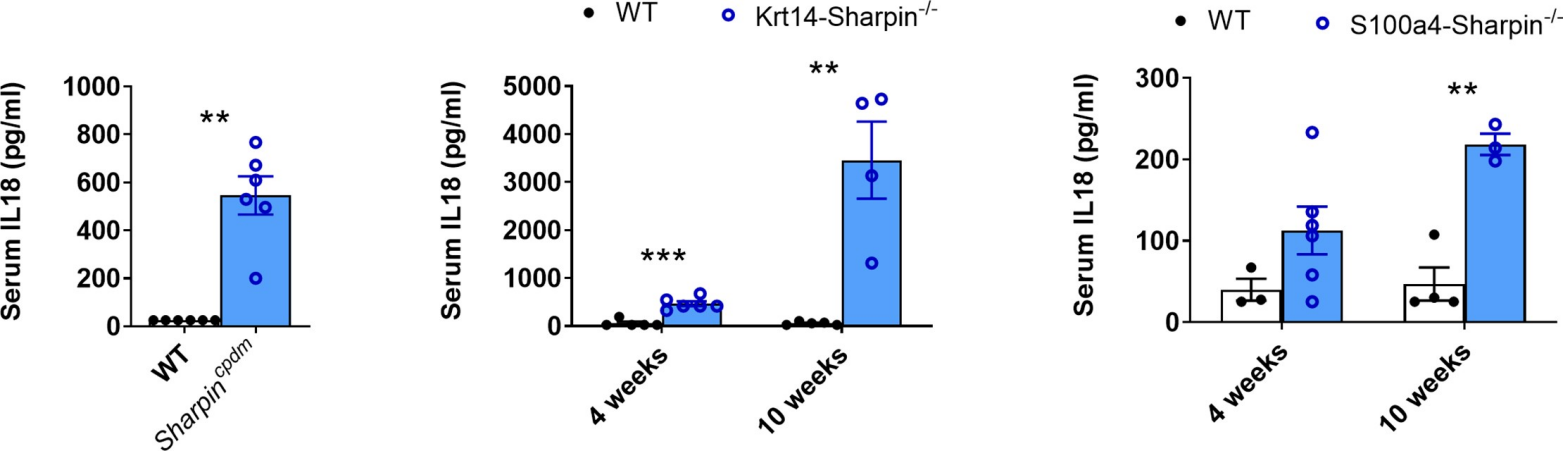
Serum IL18. Concentration of IL18 in the serum of (A) WT and *Sharpin*^*cpdm*^ mice (5–7 weeks of age, 6 mice/group), (B) *Krt14 Sharpin*^*-/-*^ and control mice (4–6 mice/group) and (C) *S100a4 Sharpin*^-/-^ and control mice (3–5 mice/group). Bars indicate the mean ± SEM. ** p < 0.01; *** p < 0.001.

IL18 induces an increase of IgE in mice [58], and increased concentration of serum IL18 has been reported in human patients with atopic dermatitis [59, 60]. We therefore determined the concentration of IL18 in the serum of control and mutant mice. There was a significant increase of IL18 in the serum of *Sharpin*^*cpdm/cpdm*^ mice compared with control mice (Fig 6). A similar increase was observed in the serum of *Krt14 Sharpin*^-/-^ and *S100a4 Sharpin*^-/-^ mice. The concentration increased with the age of the mice and the progression of the dermatitis, and was much higher in the *Krt14 Sharpin*^-/-^ mice than the other mutant strains.

### Cutaneous expression of cytokines and proteases

The spontaneous *Sharpin*^*cpdm/cpdm*^ null mice developed a persistent dermatitis associated with increased expression of T_H_2 cytokines, chitinase-like 3 protein (CHIL3), a sensitive marker of alternatively activated macrophages, and eosinophil-specific chemokines [4, 48]. The type 2 inflammation may result from lack of SHARPIN in stromal cells, immune cells, or both. To determine the effect of loss of *Sharpin* in non-immune cell types on expression of type 1 and type 2 cytokines, changes in various cytokine mRNA levels were evaluated in *CMV*, *Krt14*, and *S100a4 Sharpin*^-/-^ mice, and appropriate controls.

**Fig 7:**
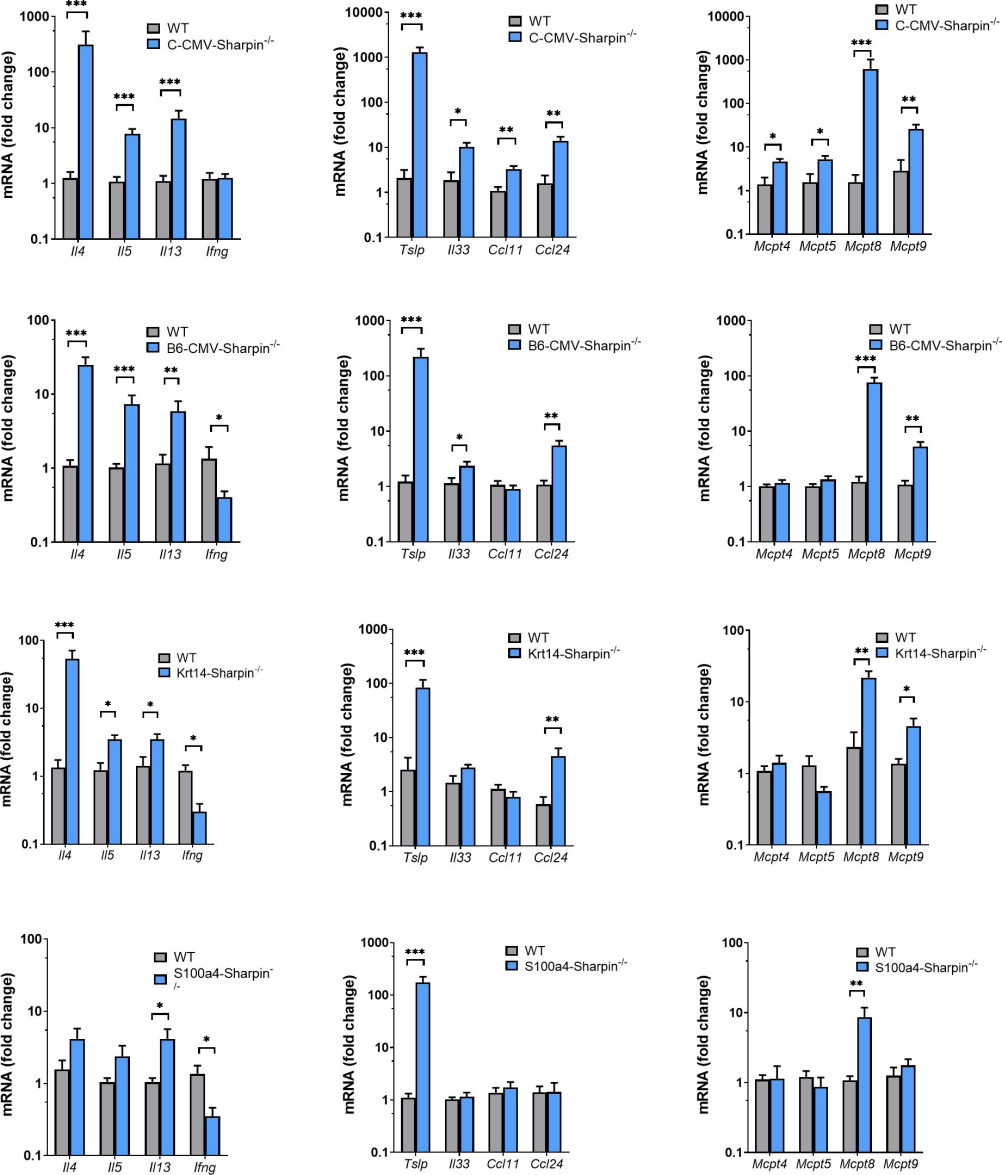
Expression of mRNA of cytokines and inflammation-related proteins in the skin of control and *C-CMV Sharpin*^-/-^, *B6-CMV Sharpin*^*-/-*^, *Krt14 Sharpin*^*-/-*^, and *S100a4 Sharpin*^-/-^ mice. The fold change of mRNA expression compared with control skin was calculated using the 2^-ΔΔCt^ method. Bars represent the mean + SEM of 6 mice/group. * p < 0.05; ** p < 0.01; *** p < 0.001.

The dermatitis in the skin of all mutant mice was associated with a highly significant (p < 0.0005) increased expression of *Chil3* consistent with type 2 inflammation (S14 Fig). This was supported by the increase of *Il4*, *Il5*, and *Il13* mRNA expression and decreased expression of *Ifng* except in the BALB-*CMV Sharpin*^-/-^ mice (Fig 7). The change in cytokine expression was greatest in the B6-*CMV Sharpin*^*-/-*^ mice and least in the *S100a4 Sharpin*^*-/-*^ mice reflecting the severity of the inflammation observed by light microscopy. These data indicate that the selective deletion of SHARPIN in stromal cells did not change the type of inflammation.

We previously reported increased expression of the epithelial cytokines thymic stromal lymphopoietin (TSLP) and IL33 in the skin of *Sharpin*^*cpdm/cpdm*^ mice [6]. There was significant increase of *Tslp* and *Il33* mRNA in the skin of *CMV Sharpin*^*-/-*^ mice, but only an increase of *Tslp* mRNA and not *Il33* mRNA in the skin of *Krt14 Sharpin*^*-/-*^ mice (Fig 7).

CCL11 (eotaxin) and CCL24 (eotaxin-2) are two eosinophil-specific chemokines that attract eosinophils into tissues and can induce their activation. We observed significantly increased expression of *Ccl11* and *Ccl24* mRNA in the skin of *B6-CMV Sharpin*^*-/-*^ mice, but only increased expression of *Ccl24* mRNA in the skin of *B6-CMV* and *Krt14 Sharpin*^*-/-*^ mice. There was no significant increase in the expression of these chemokines in the skin of *S100a4 Sharpin*^*-/-*^ mice (**Fig 7**).

There was a marked accumulation of mast cells in the skin of *Sharpin*^*cpdm/cpdm*^ mice [1]. We examined the expression of mast cell protease genes. *Mcpt4* and *5* are specifically expressed by connective tissue mast cells [61], and *Mcpt8* by basophils [62]. Surprisingly, there was a significant, but modest increase of *Mcpt4* and *Mcpt5* mRNA in the skin of *B6-CMV Sharpin*^*-/-*^ mice, but not in the skin of the other mutant mice. By contrast, the expression of *Mcpt8* mRNA was increased significantly in all mutant mice. The mast cell protease 9, encoded by *Mcpt9*, was initially reported to be selective expressed in uterine mast cells in mice [63], but is also expressed in mast cell lines derived from spleens [64]. The expression of *Mcpt9* was increased in all mutant mice except for the *S100a4 Sharpin*^*-/-*^ mice (Fig 7).

## Discussion

Correlating the specific cell types in which a gene is highly expressed and its biological function is not always straightforward. For example, the ATP-binding cassette, sub-family C (CFTR/MRP), member 6 (*Abcc6*) gene is normally highly expressed in the liver and to a lesser extent in the kidney, yet lesions, ectopic mineralization in this case, occur at other anatomical sites [65]. In this case, the ABCC6 protein acts as a transmembrane ATP efflux transporter. In other cases, genes can be highly expressed in locations where lesions occur when they are mutated, such as stearoyl-Coenzyme A desaturase 1 (*Scd1*), which regulates lipid metabolism and results in severe abnormalities in sebaceous glands when missing [66]. In the case of the *Sharpin* gene, immortalized mouse embryonic fibroblasts have been used extensively to define its biochemical functions yet the data generated has not always matched experimental results using live mice carrying spontaneous null mutations in the gene as indicated in the introduction. Primary cultures of human keratinocytes as well as mouse keratinocytes and embryonic fibroblasts have been used to study the effect of SHARPIN deficiency on TNF-induced apoptosis [12–16]. SHARPIN-deficiency leads to increased sensitivity of these cells as well as myeloid cells to TNF-induced cell death [16]. Systemic deletion of TNFR1 prevented both the dermatitis and inflammation in the liver and lungs [14, 15]. However, specific deletion of TNFR1 in keratinocytes prevented keratinocyte cell death and dermatitis, but did not ameliorate systemic inflammation [15]. Both keratinocyte and systemic cell death and inflammation are dependent on enzymatically active RIPK1 [67]. By contrast, RIPK3 and MLKL-deficiency had no effect on the dermatitis, but greatly reduced systemic inflammation [16]. This indicates cell and tissue type-specific effects and mechanisms of TNF-induced cell death caused by deficiency of SHARPIN. To begin to address the role of SHARPIN in specific cell types, a conditional *Sharpin* null mouse was created. Mutant mice were crossed with transgenic strains carrying promotors that drove *cre-*recombinase expression ubiquitously throughout the body, which would recapitulate the spontaneous *Sharpin* null allele phenotypes, or ones that expressed *cre* in keratinocytes (*Krt14*), fibroblasts (*S100a4*), or adipocytes (*Adipoq*).

Inbred, congenic, hybrid, or mixed genetic backgrounds often have major effects on single gene mutations that have a major effect on the health of the mouse which reflects effects of various modifier genes in one strain versus another [68]. Lack of large-scale standardization of strain background used to make many of the *cre*-recombinases can be a problem in some studies as was in this one. A differential effect in disease severity was observed with two different spontaneous allelic mutations in *Sharpin* where both were functional null alleles, one on a B6 substrain and the other on a BALB congenic strain, the latter being more severely affected [6]. To recapitulate this, B6- and BALB-*CMV-cre*-recombinases transgenic mice were crossed with the B6.Cg conditional *Sharpin* allele. This was initially set up using the BALB allele as it was the only one available at the time. When the B6 allele became available, it was used. Both had similar lesions at the gross, hematological, and histopathological levels; however, the B6 strain was more severely affected than the BALB, opposite that of the spontaneous null alleles of *Sharpin*. These results limited comparative studies at older ages. Since the overall disease was the same with only differences in life expectancy, identification of modifier genes could be relatively difficult. Alternatively, the effect of the *Sharpin* null mutation is quite severe and overriding more subtle differences in phenotyping. This observation allows one to be less concerned about strain background in the crosses used in this study than in other studies where background effects play a major role.

Restricting the loss of SHARPIN expression to specific cell types resulted in different phenotypes. Absence of SHARPIN expression in adipocytes did not result in an abnormal phenotype. By contrast, skin disease was severe in the ubiquitously and keratin (*Krt14*) inactivated *Sharpin* and mild in the *S100a4 Sharpin*^-/-^ cross. Verification of the expression of *S100a4-cre*-recombinase revealed that it was expressed in keratinocytes, albeit less so than for the *Krt14-cre*, but not in skin fibroblasts. While S100A4 is still sometimes published as fibroblast-specific protein [54], results shown here reveal this is not the case, rather it is expressed in the epidermal and infundibular keratinocytes. This is also the case in human skin where S100A4 is expressed in the hair follicle, Langerhans cells, and melanocytes [69, 70]. The gene/protein name has been changed to S100 calcium binding protein A4 in Mouse Genome Informatics (http://www.informatics.jax.org; 22 Jan 2020). *S100a4-cre*-recombinase expression in keratinocytes explains why lesions were present in the skin. Reduced expression of the *cre*-recombinase in the mouse skin for the *S100a4* versus the *Krt14* may explain the qualitative and quantitative differences between these two crosses. While lesions were observed in mice of both sexes at relatively the same levels, in the *S100a4 Sharpin*^-/-^ joint lesions were more severe than in the other crosses and more severe in the females. The knee joint was less often affected or less severely affected in males. Shoulder joints were more severely affected. S100A4 is involved in promoting cancer progression and metastasis, fibrosis, inflammation, immune response, neuroprotection, angiogenesis, and some common non-tumor diseases including being upregulated in cells infiltrating rheumatoid arthritis synovial tissue [71, 72]. Images on the cre-recombinase website (http://www.informatics.jax.org/recombinase/specificity?id=MGI:3712292&system=skeletal+system; 6 Feb 2020) reveal that *S100a4* is expressed in bone and bone marrow which helps to partially explain the joint specific lesions in these mice. Two factors were probably involved in the variability in arthritis between individuals. One is that inbred strains could not be used as the *cre*-recombinase transgenic mice were not on the same background or were mixed backgrounds. This alone results in phenotypic variability. The second was that since some of the crosses were very detrimental, all mice were evaluated at 6 weeks of age as that was the only age at which representative mutant mice could be obtained from all the crosses. Lesions developed more consistently with age in those that were destined to develop lesions in affected organs, especially in the joints. This work needs to be repeated in detail to determine what cell types in the bone marrow and joints that express *S100a4*. This cross provides a useful model for studying autoinflammatory arthritis. These observations combined with protein localization using immunohistochemistry support keratinocytes as the primary cell type involved in many of the lesions, particular the skin.

The dermatitis in *Sharpin*^*cpdm/cpdm*^ mice has many features in common with atopic dermatitis, but is not associated with an increase of serum IgE [3]. Indeed, previous work demonstrated that the dermatitis develops in the absence of functional B and T cells [6]. Mutant *Sharpin*^*cpdm/cpdm*^ mice have decreased or undetectable serum IgG, IgA, and IgE and a modest increase of serum IgM [3]. This indicates a defect in isotype switching consistent with the impairment of NFkB signaling upon activation of B cells with anti-CD40 [12–14]. Remarkably, selective deletion of *Sharpin* in keratinocytes is sufficient to recapitulate the dermatitis of mice with the spontaneous *Sharpin* mutation, and this was associated with a marked increase of serum IgE as the B cells in *Krt14 Sharpin*^*-/-*^ mice were able to undergo isotype switching. The mechanism that underlies the increase of serum IgE is not entirely clear, but it likely involves increased secretion of IL18. The serum concentration of IL18 was increased dramatically in *Krt14 Sharpin*^*-/-*^ mice. Transgenic mice with overexpression of IL18 in the epidermis develop a type 2 inflammatory dermatitis and increase of serum IgE [73]. In the absence of STAT6, required for signaling through the IL4 and IL13 receptor, these mice had undetectable IgE, but developed a similar dermatitis indicating that the IgE was not necessary for the development of the dermatitis. A direct role for IL18 in the increase of IgE is further supported by the observation that daily injection of IL18 induces an increase of serum IgE in an IL4-dependent manner [58]. An increase of serum IL18 has also been observed in human patients with atopic dermatitis and it has been suggested that IL18 can serve as a biomarker of disease severity [74].

TSLP and IL33 can be secreted by epithelial cells upon damage or stress and induce local inflammation through the activation of dendritic cells, type 2 innate lymphoid cells (ILC2), basophils, and mast cells [75]. Both *Tslp* and *Il33* mRNA were increased in the skin of BALB- and *B6-CMV Sharpin*^*-/-*^ mice, but only *Tslp* mRNA was increased in the skin of *Krt14 Sharpin*^*-/-*^ and *S100a4 Sharpin*^*-/-*^ mice. TSLP and IL33 both play a role in the induction of atopic dermatitis-like skin lesions following topical application of the vitamin D analogue calcipotriol [76, 77], and overexpression of TSLP and IL33 in the epidermis of transgenic mice induced a type 2 inflammatory response with accumulation of mast cells, eosinophils, and an increase of serum IgE [78, 79]. The expression of TSLP and IL33 is increased in the skin of human patients with atopic dermatitis [80, 81]. The dermatitis in SHARPIN-deficient mice is associated with apoptosis of keratinocytes [1]. The apoptosis and dermatitis were inhibited by deletion of TNFR1 [15, 16, 82]. This suggests that keratinocyte damage in the absence of SHARPIN may lead to release of TSLP and IL33, which initiate the dermatitis through the activation of ILC2 and basophils.

CCL11 and CCL24 are both ligands of the chemokine receptor CCR3 expressed on eosinophils and are involved in the recruitment and activation of eosinophils [83]. They are both secreted by fibroblasts upon stimulation with IL4 and IL13, but they have overlapping but distinct expression patterns and functions. Using genetically engineered mice, it was demonstrated that CCL24 had a dominant role in the recruitment of eosinophils into the airways of mice in an asthma model [84]. The expression of CCL24 was more consistently and strongly increased compared with CCL11 in the skin of mutant mice suggesting a more important role for CCL24 in the dermatitis of SHARPIN-deficient mice.

The dermatitis in mutant *Sharpin*^*cpdm/cpdm*^ mice is characterized by a marked increase of the number of mast cells. It was therefore surprising that the expression of mast cell proteases *Mcpt4* and *Mcpt5*, expressed in dermal connective tissue mast cells [61], was not or only minimally increased in the skin of mutant mice. However, treatment of bone marrow-derived mast cells with IL4 decreased the expression of *Mcpt4* [85], suggesting that the expression of mast cell proteases is affected by the cytokines in a type 2 inflammatory environment. The mast cell protease 9 was initially reported to be exclusively expressed in mast cells isolated from the uterus in mice [63]. However, it can be expressed by other mast cells [64], and we show here that its expression is increased in the skin of mutant mice with dermatitis. The mast cell protease 8 is used as a marker of basophils based on the exclusive expression in these cells [62], although a report suggests that it is also expressed in granulocyte-macrophage progenitor cells [86]. Nevertheless, in the skin *Mcpt8* expression is associated with basophils, and the marked increased expression of *Mcpt8* suggests an increased number of basophils in the skin. Both TSLP and IL33 enhance the production and activation of basophils [87, 88].

Spontaneous *Sharpin* mutations that arose in laboratory mice [2] provided useful models to dissect linear ubiquitin chain assembly complex (LUBAC) which is required for activation of the NFkB signaling pathway [12–14, 89] and they continue to do so [16, 27, 30, 34, 90]. The conditional *Sharpin* null allele, which is publicly available at The Jackson Laboratory, provides a new tool to further investigate this molecular pathway. Refining the mouse model, especially using the *S100a4-cre*-recombinase transgenic mouse, provides tools to investigate autoinflammatory arthritis and atopic dermatitis.

## Supporting information

S1 Fig Cre-recombinase expression for the different promotors used. *Krt14-cre* was expressed throughout the epidermis and hair follicles (A). *S100A4-cre*, which was expected to be limited to fibroblasts, was strongly expressed in keratinocytes of the epidermis and parts of the hair follicle (B). *Adipoqu-cre* expression was limted to white and brown adipose tissue (C).
(TIF)

S2 Fig Change in body weight by cross. Males were consistently heavier than females. Mice with severe skin or skin and visceral lesions had moderate to severe weight loss as they aged.
(TIF)

S3 Fig Peripheral blood values. White blood cells in peripheral blood of wild-type (WT), *Krt14 Sharpin*^*-/-*^(Krt14-Sharpin), and *S100a4 Sharpin*^-/-^ (S100a4-Sharpin) mice. Bars represent the mean + SEM. * P < 0.05; ** P < 0.01.
(TIF)

S4 Fig Limiting ridge and forestomach histology. The normal forestomach of mice is lined by stratified squamous epithelium. The epithelium forms a papillomatous structure, stratified squamous epithelium on a fibrovascular stalk, called the limiting ridge that forms a junction with the glandular stomach. Female mice, 6 weeks of age, carrying any of the *cre*-recombinase transgenes but no or only one copy of the conditional *Sharpin* gene were normal (A). Mice carrying one or two copies of the ubiquitously expressing *CMV-cre* and homozygous for the conditional *Sharpin* gene (*Sharpin*^*-/-*^) (C, D) had lesions identical to the spontaneous *Sharpin*^*cpdm/cpdm*^ mice (B). The squamous epithelia were moderately to severely acanthotic with orthokeratotic hyperkeratosis and moderate apoptosis of keratinocytes, very similar to epidermal changes in affected mice. While KRT14 is expressed in the forestomach by immunohistochemistry (data not shown) similar to where SHARPIN is expressed (**Fig 2N**) the forestomach was normal in the *Krt14-cre* mice (E). Mice carrying the *S100a4-cre* (F) or *Adipoq-cre* (G) all had normal forestomach anatomy. Low magnification 100X, high magnification 400X.
(TIF)

S5 Fig Liver histology. Female mice, 6 weeks of age, carrying any of the *cre*-recombinase transgenes but no or only one copy of the conditional *Sharpin* gene were normal (A). Mice carrying one or two copies of the ubiquitously expressing *CMV-cre* and homozygous for the conditional *Sharpin* gene (*Sharpin*^-/-^) (C, D) had lesions identical to the spontaneous *Sharpin*^*cpdm/cpdm*^ mice (B). A mixed inflammatory cell infiltrate was present surrounding large hepatic veins and portal triads. Fibrosis was a feature of the inflammation around large veins. Mice carrying the *Krt14-cre* (E), *S100a4-cre* (F), or *Adipoq-cre* (G) all had normal livers. Low magnification 40X, high magnification 40X.
(TIF)

S6 Fig Lung histology. Female mice, 6 weeks of age, carrying any of the *cre*-recombinase transgenes but no or only one copy of the conditional *Sharpin* gene were normal (A). Mice carrying one or two copies of the ubiquitously expressing *CMV-cre* and homozygous for the conditional *Sharpin* gene (*Sharpin*^*-/-*^) (C, D) had lesions identical to the spontaneous *Sharpin*^*cpdm/cpdm*^ mice (E). There was a mixed inflammatory cell infiltrate around the bronchioles and pulmonary veins. Mice that were *Krt14* (E), *S100a4* (F), or *Adipoq Sharpin*^*-/-*^ (G) all had normal lungs. Low magnification 40X, high magnification 400X.
(TIF)

S7 Fig Shoulder joint. Female mice, 6 weeks of age, carrying any of the *cre*-recombinase transgenes but no or only one copy of the conditional *Sharpin* gene were normal (A). Mice carrying one or two copies of the ubiquitously expressing *CMV-cre* and homozygous for the conditional *Sharpin* gene (*Sharpin*^*-/-*^) (C, D) had lesions similar to but less severe than in the spontaneous *Sharpin*^*cpdm/cpdm*^ mice (B). The soft tissue surrounding the joint capsule had mild infiltration by granulocytes. Granulocytes and fibrin were present within the joint space. Severity varied between individuals with some males having more severe lesions. *Krt14 Sharpin*^*-/-*^ mice were normal (E). *S100a4 Sharpin*^*-/-*^ consistently had severe lesions (F). *Adipoq Sharpin*^*-/-*^ mice were unaffected. Low magnification 40X, high magnification 400X.
(TIF)

S8 Fig Knee joint histology. Female mice, 6 weeks of age, carrying any of the *cre*-recombinase transgenes but no or only one copy of the conditional *Sharpin* gene were normal (A). Mice carrying one or two copies of the ubiquitously expressing *CMV-cre* and homozygous for the conditional *Sharpin* gene (*Sharpin*^*-/-*^) (C, D) had lesions identical to the spontaneous *Sharpin*^*cpdm/cpdm*^ mice (B). The soft tissue surrounding the joint capsule had mild infiltration by granulocytes. Granulocytes were present within the joint space but were few in number. *Krt14* (E) and *Adipoq Sharpin*^-/-^ (G) joints were unaffected. However, joint lesions were more prominent and severe in the *S100a4-cre* mice (F) but less so than in the knee or temporomandibular joints. Low magnification 40X, high magnification 400X.
(TIF)

S9 Fig Middle ear histology. Female mice, 6 weeks of age, carrying any of the *cre*-recombinase transgenes but no or only one copy of the conditional *Sharpin*^*-/-*^ gene were normal (A) having middle ears with no evidence of inflammation. By contrast, mice homozygous for the spontaneous *Sharpin*^*cpdm/cpdm*^ mutation consistently had moderate to severe mixed inflammatory cells in the middle ear and surrounding soft tissues (B). Mice carrying one or two copies of the ubiquitously expressing *CMV-cre* and homozygous for the conditional *Sharpin* gene (*Sharpin*^*-/-*^) (C, D) also had middle ear inflammation. Mice carrying the *Krt14* (E), *S100a4* (F), or *Adipoq Sharpin*^*-/-*^ (G) all had normal, unaffected, middle ears. Low magnification 40X, high magnification 400x.
(TIF)

S10 Fig Peyer’s patch histology. Female mice, 6 weeks of age, carrying any of the *cre*-recombinase transgenes but no or only one copy of the conditional *Sharpin*^*-/-*^ gene were normal (A) having Peyer’s patches in their small intestines. Mice carrying one or two copies of the ubiquitously expressing *CMV-cre* and homozygous for the conditional *Sharpin* gene (*Sharpin*^*-/-*^) (C, D) had lesions identical to the spontaneous *Sharpin*^*cpdm/cpdm*^ mice (B). At this age either there was no evidence of Peyer’s patches (C, D) or remnants effaced by eosinophils (B, high mag). Mice carrying the *Krt14* (E), *S100a4* (F), or *Adipoq Sharpin*^*-/-*^ (G) all had normal Peyer’s patches. Low magnification 40X, high magnification 400X.
(TIF)

S11 Fig Spleen histology. Female mice, 6 weeks of age, carrying any of the *cre*-recombinase transgenes but no or only one copy of the conditional *Sharpin* gene were normal (A). Mice carrying one or two copies of the ubiquitously expressing *CMV-cre* and homozygous for the conditional *Sharpin* gene (*Sharpin*^*-/-*^) (C, D) had lesions identical to the spontaneous *Sharpin*^*cpdm/cpdm*^ mice (B). In all 3 of these groups the spleen had severely disrupted white pulp microarchitecture. There was no separate T- and B-cell areas, lack of follicles, marginal zone, and follicular dendritic cells [91]. Mice carrying the *Krt14* (E), *S100a4* (F), or *Adipoq* (G) all had normal spleens. Low magnification 40X, high magnification 400X.
(TIF)

S12 Fig Cervical lymph node histology. Female mice, 6 weeks of age, carrying any of the *cre*-recombinase transgenes but no or only one copy of the conditional *Sharpin* gene were normal (A). Mice homozygous for the spontaneous chronic proliferative dermatitis (*Sharpin*^*cpdm/cpdm*^) null mutation had marked lymphoid depletion with effacement of the lymph node by eosinophils (B). Mice carrying one or two copies of the ubiquitously expressing *CMV-cre* on either the BALB (C) or B6 (D) background and homozygous for the conditional *Sharpin* gene (*Sharpin*^*-/-*^) had lesions identical to the spontaneous *Sharpin*^*cpdm/cpdm*^ mice (B). All lymph nodes throughout the body were similarly affected. The cortex and follicles were missing, effaced by a population of granulocytes that were primarily eosinophils. Occasionally areas of the medulla had fibrin deposition and necrosis in the regions where follicles are usually found, as shown for *Sharpin*^*cpdm/cpdm*^ (B, higher magnification). Mice carrying the *Krt14* (E), *S100a4* (F), or *Adipoq Sharpin*^*-/-*^ (G) all had normal lymph nodes throughout the body. Low magnification 40X, high magnification 400X.
(TIF)

S13 Fig Serum immunoglobulins in 5–7 week old WT and *Sharpin*^*cpdm/cpdm*^ mice. Bars represent the mean ± SE of 6 mice/group. * p < 0.05.
(TIF)

S14 Fig Expression of mRNA of *Chil3* in wild type (WT) and the indicated mutant mice. Bars represent the mean + SEM of fold change in gene expression in mutant vs. WT mice. *** P < 0.001.
(TIF)

S15 Fig Original uncropped and unadjusted images underlying results reported in Fig 1. (TIF)

## Abbreviations

cIAP1/2: Cellular Inhibitor of Apoptosis Protein 1/2
cpdm: chronic proliferative dermatitis mutant mouse allele of *Sharpin*
*Cre*-recombinase: a tyrosine recombinase: enzyme: derived from the: P1 bacteriophage
Cyc1: cytochrome c-1
flt: flippase
frt: flippase recognition target
Loxp: locus of crossover in P1
LUBAC: linear ubiquitination assembly complex
Maf1: MAF1 homolog, negative regulator of RNA polymerase III
neo: neomycin resistance
PGK-neo: phosphoglycerine kinase-neomycin resistance
PTEN: phosphatase and tensin homolog
RIPK1: receptor (TNFRSF)-interacting serine-threonine kinase 1
*Sharpin*/SHARPIN: (*gene*/protein) SHANK-associated RH domain interactor
Sharpin^-/^: B6(Cg)-*Tyr*^*c-2J*^ *Sharpin*^*tm1Sun*^/Sun
TNFR1: tumor necrosis factor receptor superfamily, member 1a
Tyr: tyrosinase gene
UBL: ubiquitin-like domain
