# Supplementary figures and images for: Keratinocyte-specific deletion of SHARPIN induces atopic dermatitis-like inflammation in mice
Source: PLoS One. 2020 Jul 20;15(7):e0235295. doi: 10.1371/journal.pone.0235295 (PMC7371178; doi:10.1371/journal.pone.0235295)

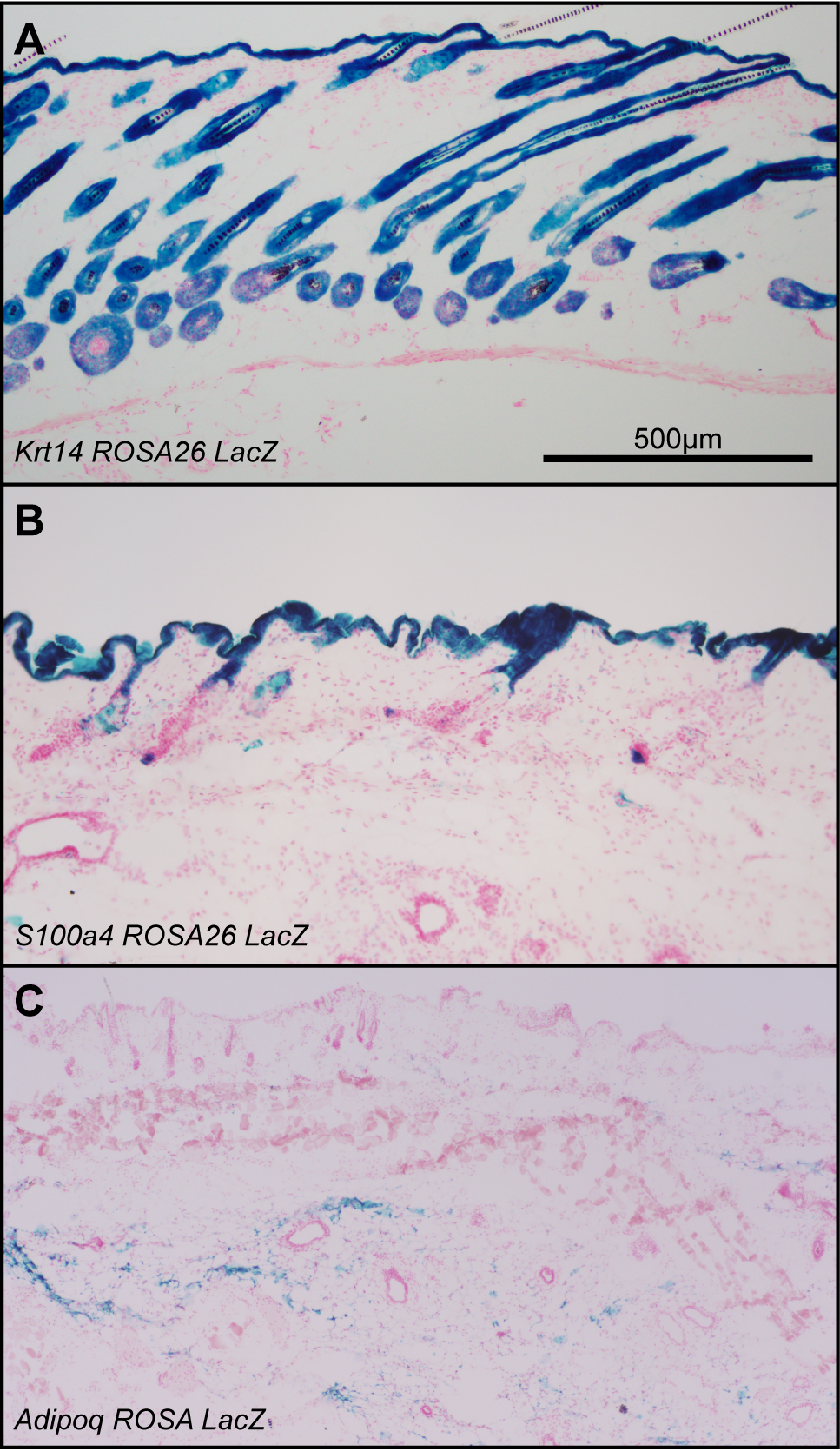

Supplement: S1 Fig — Krt14-cre was expressed throughout the epidermis and hair follicles (A). S100A4-cre, which was expected to be limited to fibroblasts, was strongly expressed in keratinocytes of the epidermis and parts of the hair follicle (B). Adipoqu-cre expression was limted to white and brown adipose tissue (C). (TIF) [file pone.0235295.s001.tif]

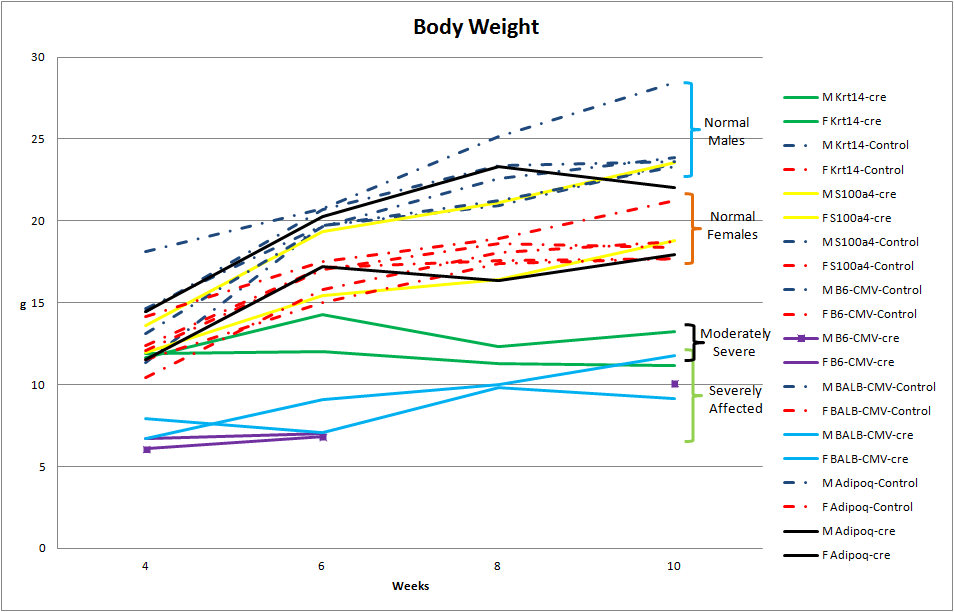

Supplement: S2 Fig — Males were consistently heavier than females. Mice with severe skin or skin and visceral lesions had moderate to severe weight loss as they aged. (TIF) [file pone.0235295.s002.tif]

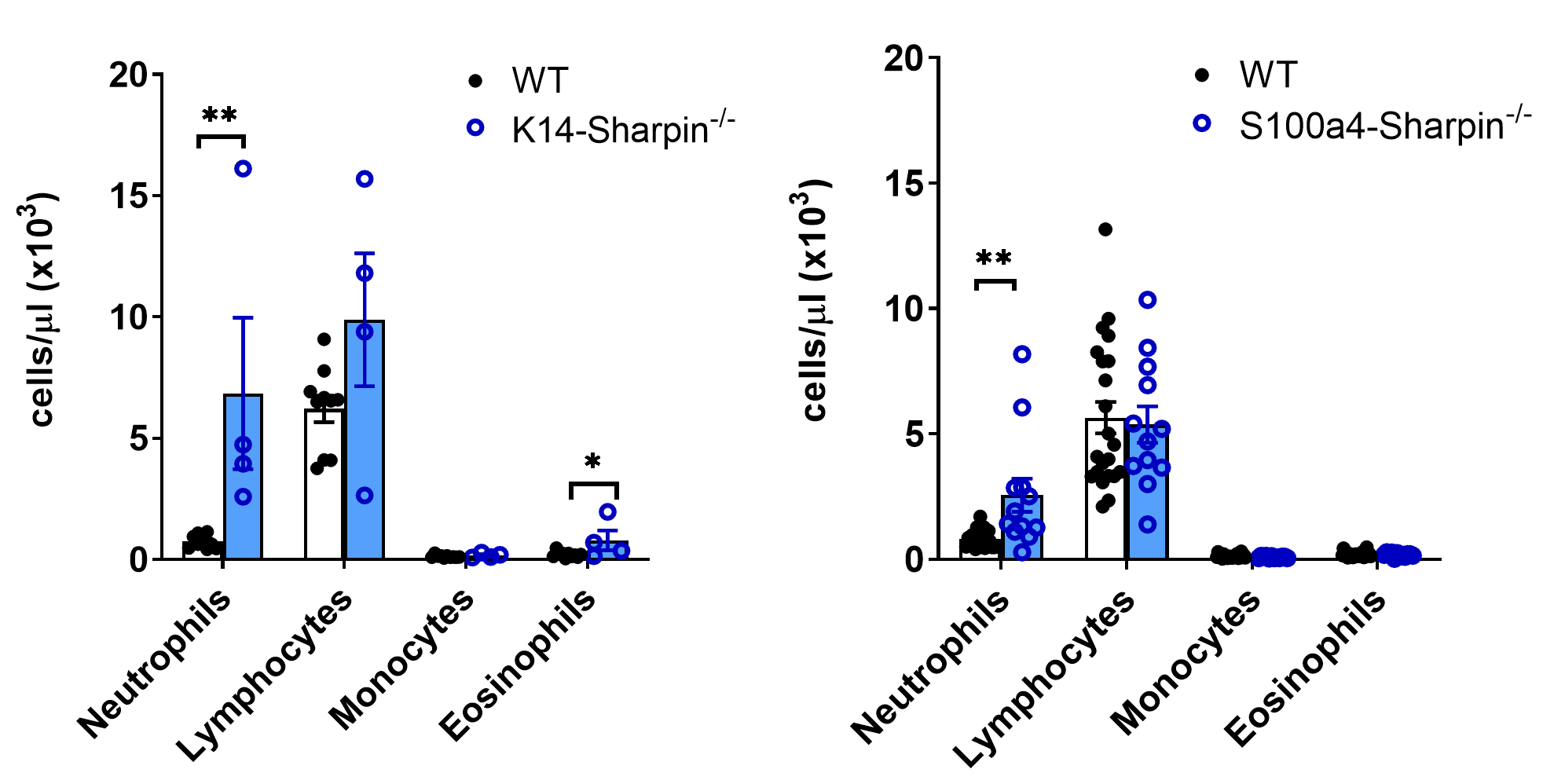

Supplement: S3 Fig — White blood cells in peripheral blood of wild-type (WT), Krt14 Sharpin-/-(Krt14-Sharpin), and S100a4 Sharpin-/- (S100a4-Sharpin) mice. Bars represent the mean + SEM. * P < 0.05; ** P < 0.01. (TIF) [file pone.0235295.s003.tif]

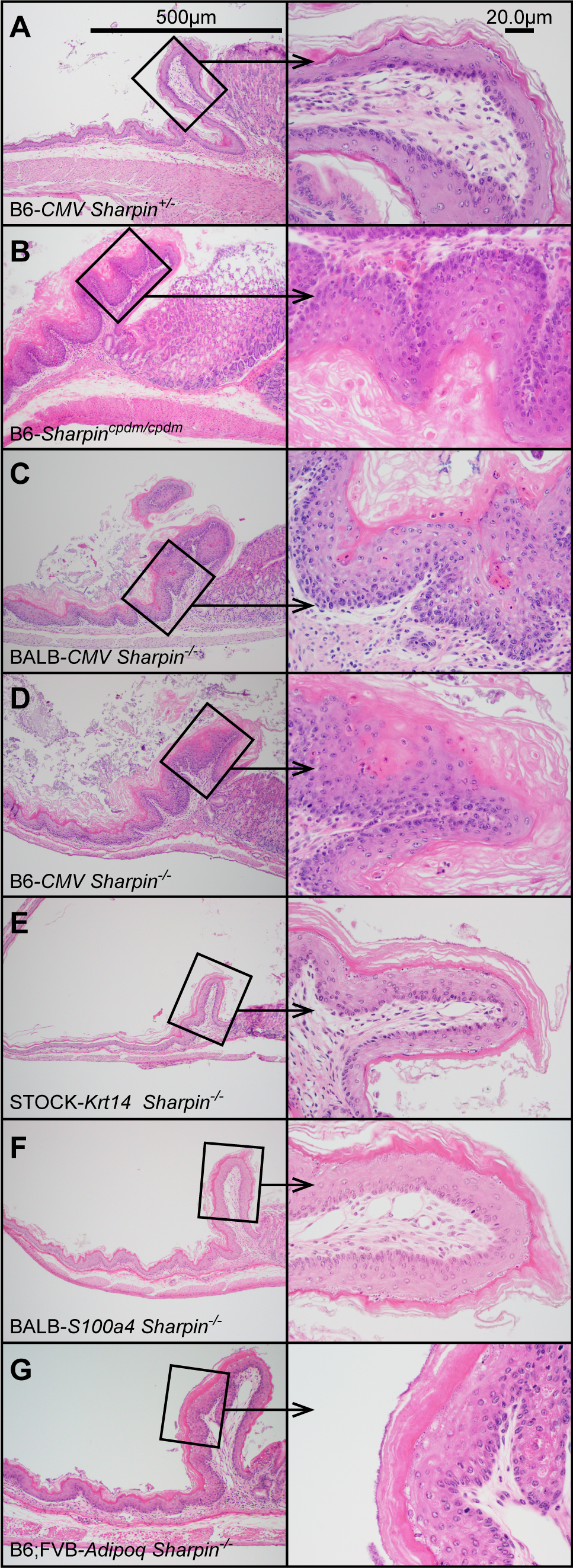

Supplement: S4 Fig — The normal forestomach of mice is lined by stratified squamous epithelium. The epithelium forms a papillomatous structure, stratified squamous epithelium on a fibrovascular stalk, called the limiting ridge that forms a junction with the glandular stomach. Female mice, 6 weeks of age, carrying any of the cre-recombinase transgenes but no or only one copy of the conditional Sharpin gene were normal (A). Mice carrying one or two copies of the ubiquitously expressing CMV-cre and homozygous for the conditional Sharpin gene (Sharpin-/-) (C, D) had lesions identical to the spontaneous Sharpincpdm/cpdm mice (B). The squamous epithelia were moderately to severely acanthotic with orthokeratotic hyperkeratosis and moderate apoptosis of keratinocytes, very similar to epidermal changes in affected mice. While KRT14 is expressed in the forestomach by immunohistochemistry (data not shown) similar to where SHARPIN is expressed (Fig 2N) the forestomach was normal in the Krt14-cre mice (E). Mice carrying the S100a4-cre (F) or Adipoq-cre (G) all had normal forestomach anatomy. Low magnification 100X, high magnification 400X. (TIF) [file pone.0235295.s004.tif]

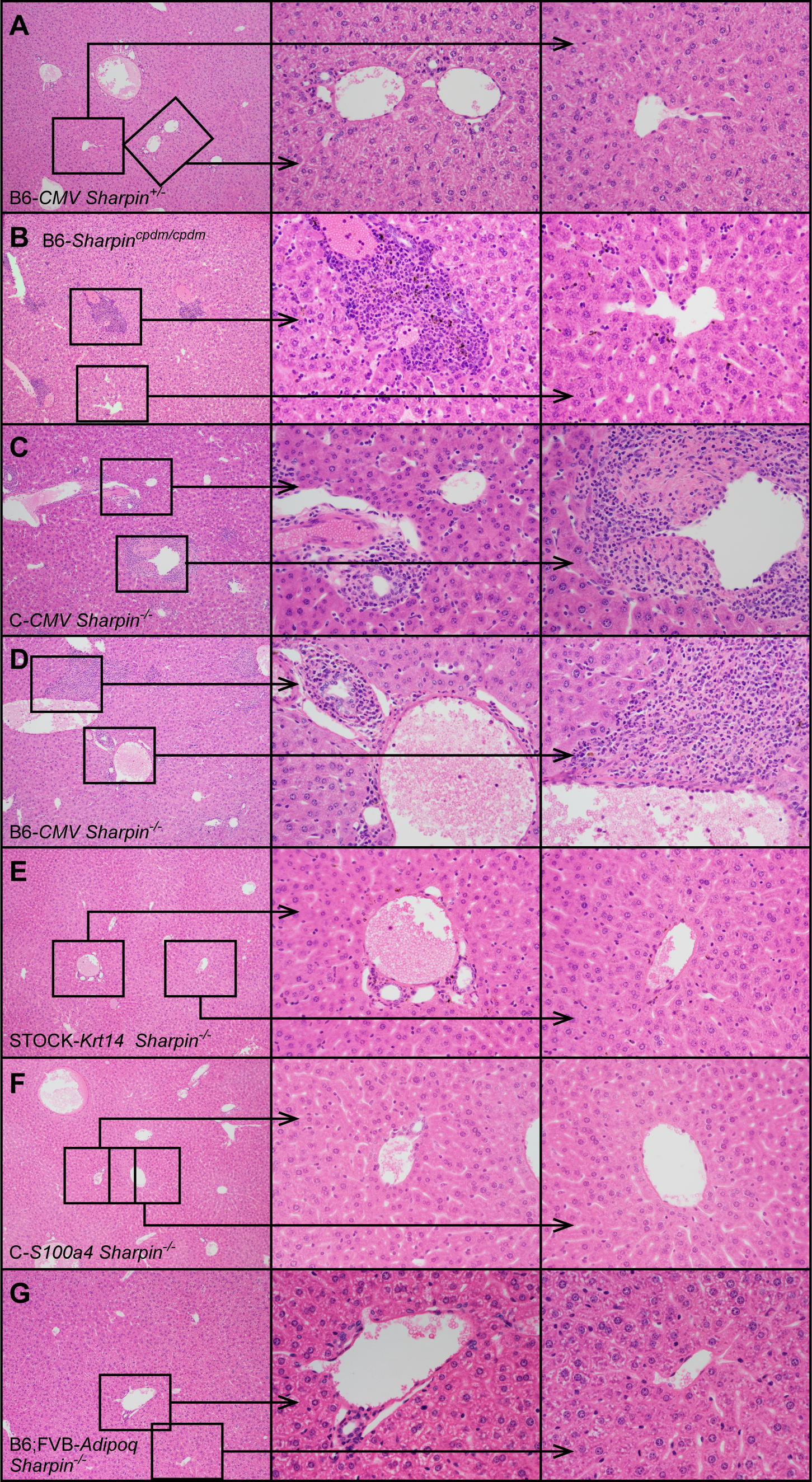

Supplement: S5 Fig — Female mice, 6 weeks of age, carrying any of the cre-recombinase transgenes but no or only one copy of the conditional Sharpin gene were normal (A). Mice carrying one or two copies of the ubiquitously expressing CMV-cre and homozygous for the conditional Sharpin gene (Sharpin-/-) (C, D) had lesions identical to the spontaneous Sharpincpdm/cpdm mice (B). A mixed inflammatory cell infiltrate was present surrounding large hepatic veins and portal triads. Fibrosis was a feature of the inflammation around large veins. Mice carrying the Krt14-cre (E), S100a4-cre (F), or Adipoq-cre (G) all had normal livers. Low magnification 40X, high magnification 40X. (TIF) [file pone.0235295.s005.tif]

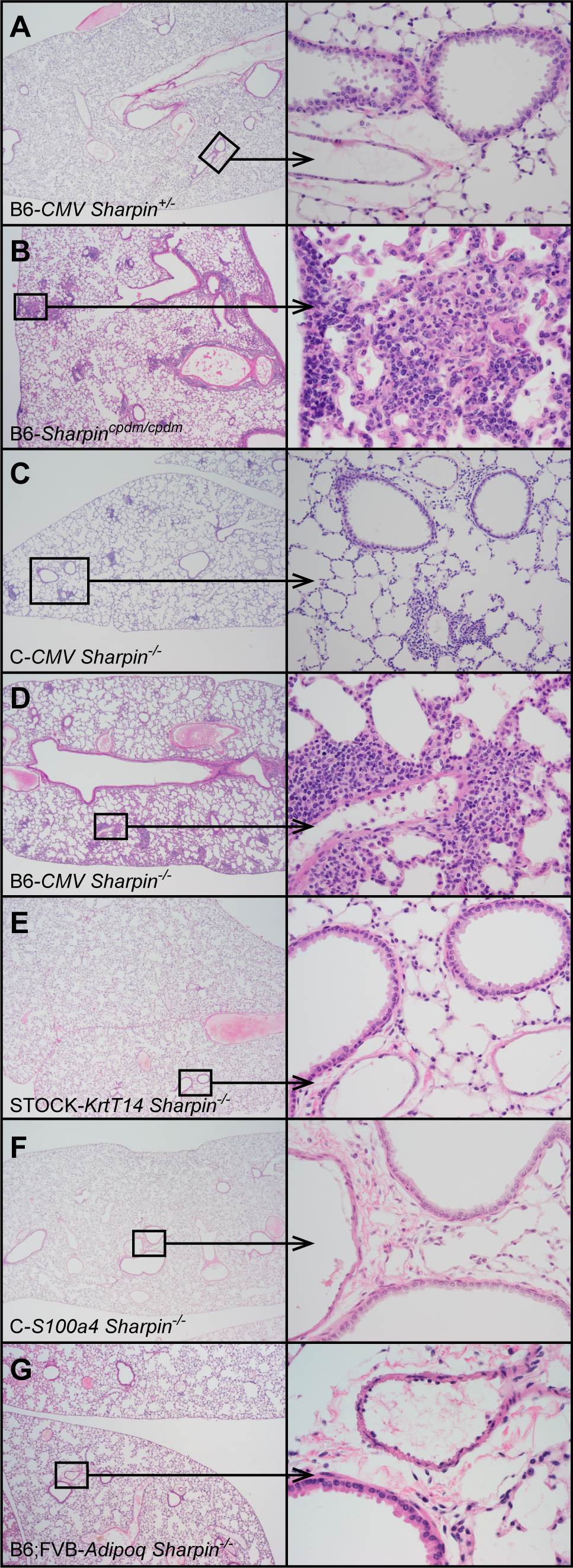

Supplement: S6 Fig — Female mice, 6 weeks of age, carrying any of the cre-recombinase transgenes but no or only one copy of the conditional Sharpin gene were normal (A). Mice carrying one or two copies of the ubiquitously expressing CMV-cre and homozygous for the conditional Sharpin gene (Sharpin-/-) (C, D) had lesions identical to the spontaneous Sharpincpdm/cpdm mice (E). There was a mixed inflammatory cell infiltrate around the bronchioles and pulmonary veins. Mice that were Krt14 (E), S100a4 (F), or Adipoq Sharpin-/- (G) all had normal lungs. Low magnification 40X, high magnification 400X. (TIF) [file pone.0235295.s006.tif]

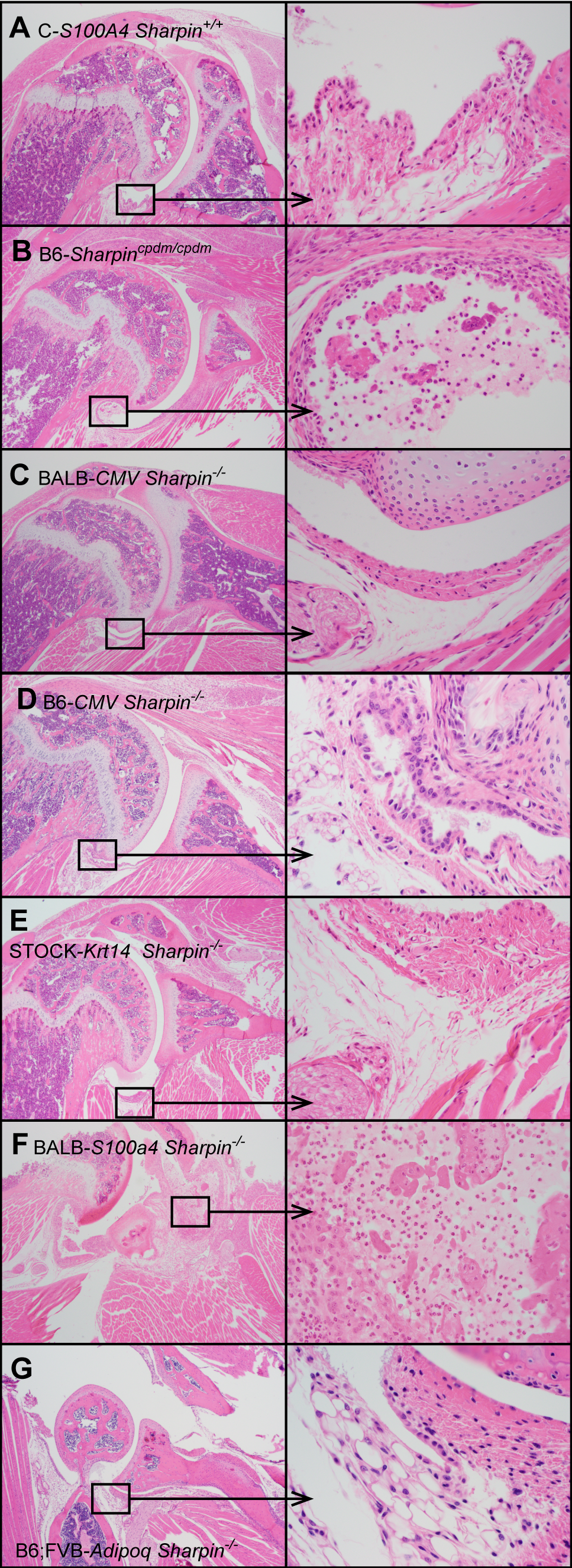

Supplement: S7 Fig — Female mice, 6 weeks of age, carrying any of the cre-recombinase transgenes but no or only one copy of the conditional Sharpin gene were normal (A). Mice carrying one or two copies of the ubiquitously expressing CMV-cre and homozygous for the conditional Sharpin gene (Sharpin-/-) (C, D) had lesions similar to but less severe than in the spontaneous Sharpincpdm/cpdm mice (B). The soft tissue surrounding the joint capsule had mild infiltration by granulocytes. Granulocytes and fibrin were present within the joint space. Severity varied between individuals with some males having more severe lesions. Krt14 Sharpin-/- mice were normal (E). S100a4 Sharpin-/- consistently had severe lesions (F). Adipoq Sharpin-/- mice were unaffected. Low magnification 40X, high magnification 400X. (TIF) [file pone.0235295.s007.tif]

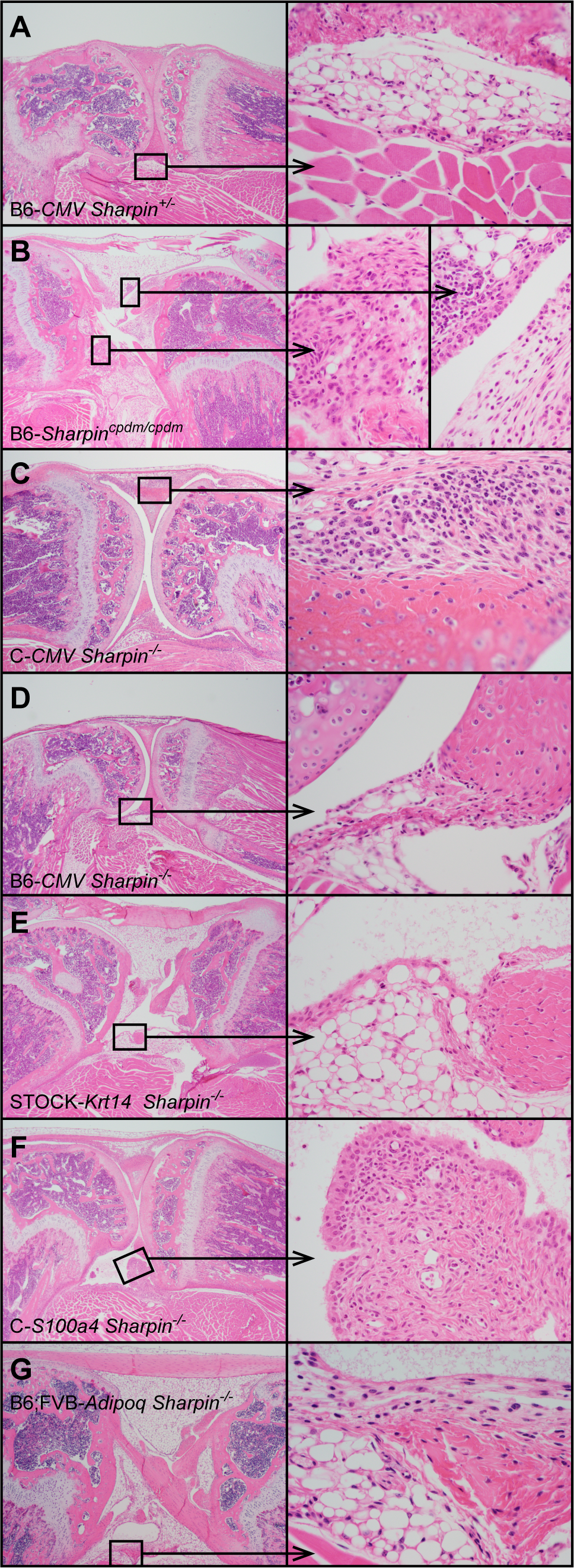

Supplement: S8 Fig — Female mice, 6 weeks of age, carrying any of the cre-recombinase transgenes but no or only one copy of the conditional Sharpin gene were normal (A). Mice carrying one or two copies of the ubiquitously expressing CMV-cre and homozygous for the conditional Sharpin gene (Sharpin-/-) (C, D) had lesions identical to the spontaneous Sharpincpdm/cpdm mice (B). The soft tissue surrounding the joint capsule had mild infiltration by granulocytes. Granulocytes were present within the joint space but were few in number. Krt14 (E) and Adipoq Sharpin-/- (G) joints were unaffected. However, joint lesions were more prominent and severe in the S100a4-cre mice (F) but less so than in the knee or temporomandibular joints. Low magnification 40X, high magnification 400X. (TIF) [file pone.0235295.s008.tif]

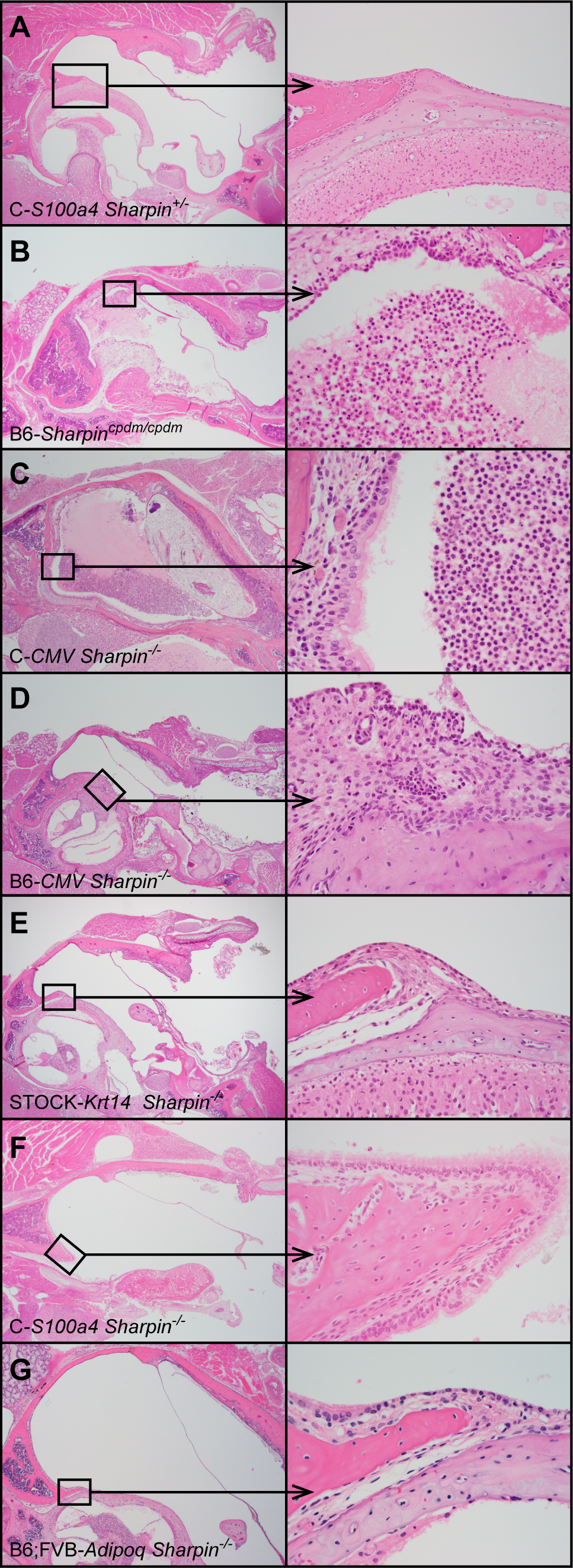

Supplement: S9 Fig — Female mice, 6 weeks of age, carrying any of the cre-recombinase transgenes but no or only one copy of the conditional Sharpin-/- gene were normal (A) having middle ears with no evidence of inflammation. By contrast, mice homozygous for the spontaneous Sharpincpdm/cpdm mutation consistently had moderate to severe mixed inflammatory cells in the middle ear and surrounding soft tissues (B). Mice carrying one or two copies of the ubiquitously expressing CMV-cre and homozygous for the conditional Sharpin gene (Sharpin-/-) (C, D) also had middle ear inflammation. Mice carrying the Krt14 (E), S100a4 (F), or Adipoq Sharpin-/- (G) all had normal, unaffected, middle ears. Low magnification 40X, high magnification 400x. (TIF) [file pone.0235295.s009.tif]

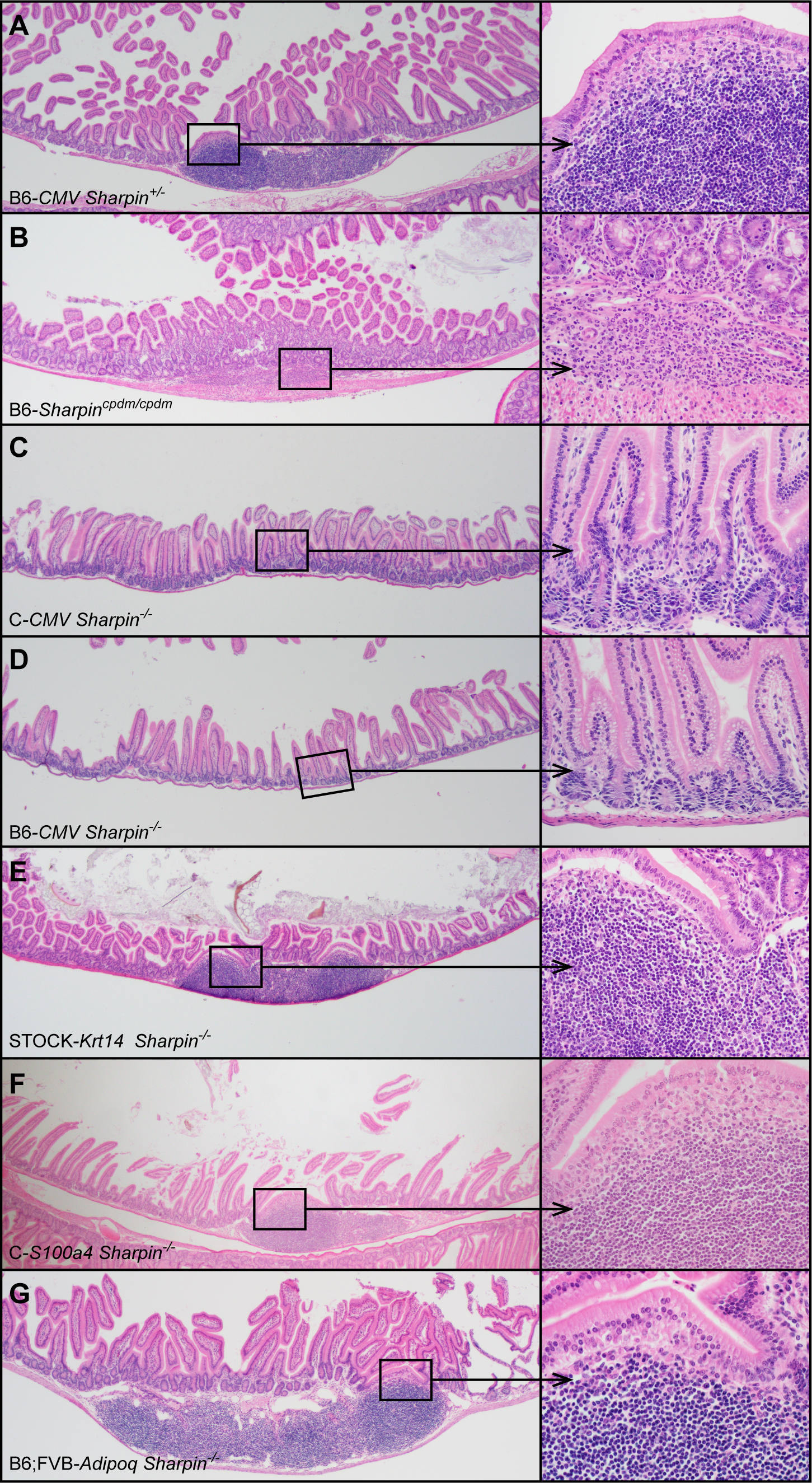

Supplement: S10 Fig — Female mice, 6 weeks of age, carrying any of the cre-recombinase transgenes but no or only one copy of the conditional Sharpin-/- gene were normal (A) having Peyer’s patches in their small intestines. Mice carrying one or two copies of the ubiquitously expressing CMV-cre and homozygous for the conditional Sharpin gene (Sharpin-/-) (C, D) had lesions identical to the spontaneous Sharpincpdm/cpdm mice (B). At this age either there was no evidence of Peyer’s patches (C, D) or remnants effaced by eosinophils (B, high mag). Mice carrying the Krt14 (E), S100a4 (F), or Adipoq Sharpin-/- (G) all had normal Peyer’s patches. Low magnification 40X, high magnification 400X. (TIF) [file pone.0235295.s010.tif]

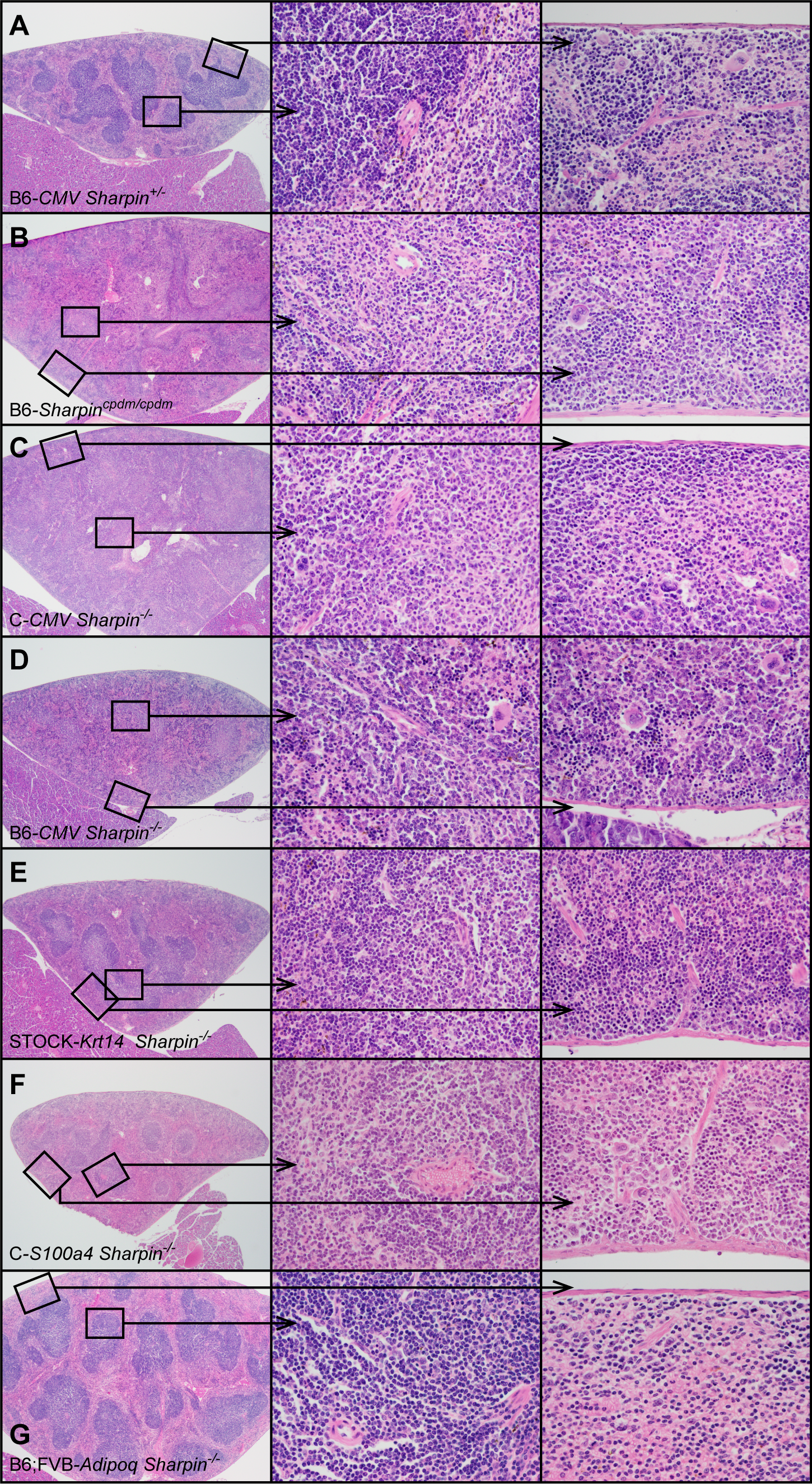

Supplement: S11 Fig — Female mice, 6 weeks of age, carrying any of the cre-recombinase transgenes but no or only one copy of the conditional Sharpin gene were normal (A). Mice carrying one or two copies of the ubiquitously expressing CMV-cre and homozygous for the conditional Sharpin gene (Sharpin-/-) (C, D) had lesions identical to the spontaneous Sharpincpdm/cpdm mice (B). In all 3 of these groups the spleen had severely disrupted white pulp microarchitecture. There was no separate T- and B-cell areas, lack of follicles, marginal zone, and follicular dendritic cells [91]. Mice carrying the Krt14 (E), S100a4 (F), or Adipoq (G) all had normal spleens. Low magnification 40X, high magnification 400X. (TIF) [file pone.0235295.s011.tif]

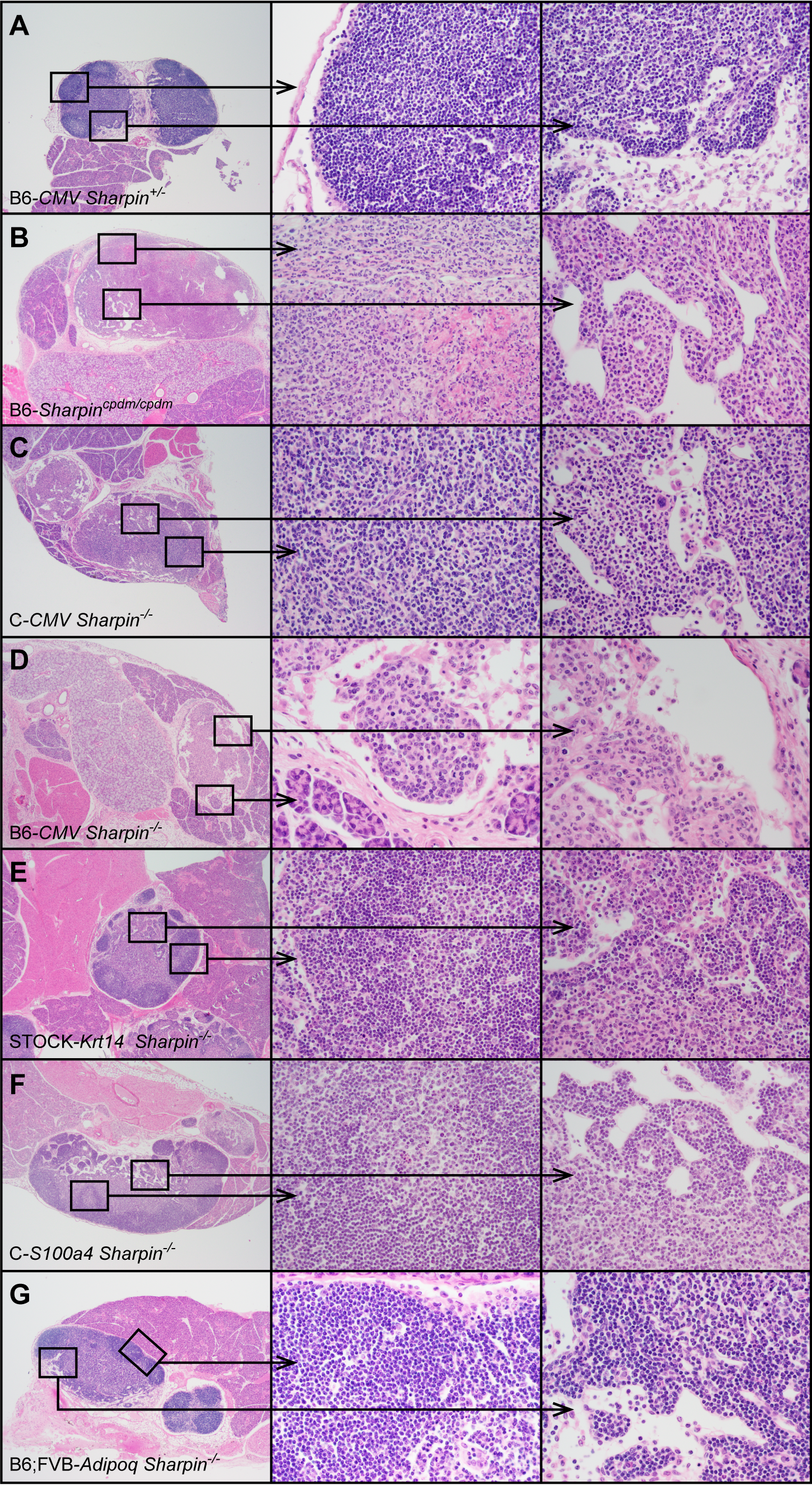

Supplement: S12 Fig — Female mice, 6 weeks of age, carrying any of the cre-recombinase transgenes but no or only one copy of the conditional Sharpin gene were normal (A). Mice homozygous for the spontaneous chronic proliferative dermatitis (Sharpincpdm/cpdm) null mutation had marked lymphoid depletion with effacement of the lymph node by eosinophils (B). Mice carrying one or two copies of the ubiquitously expressing CMV-cre on either the BALB (C) or B6 (D) background and homozygous for the conditional Sharpin gene (Sharpin-/-) had lesions identical to the spontaneous Sharpincpdm/cpdm mice (B). All lymph nodes throughout the body were similarly affected. The cortex and follicles were missing, effaced by a population of granulocytes that were primarily eosinophils. Occasionally areas of the medulla had fibrin deposition and necrosis in the regions where follicles are usually found, as shown for Sharpincpdm/cpdm (B, higher magnification). Mice carrying the Krt14 (E), S100a4 (F), or Adipoq Sharpin-/- (G) all had normal lymph nodes throughout the body. Low magnification 40X, high magnification 400X. (TIF) [file pone.0235295.s012.tif]

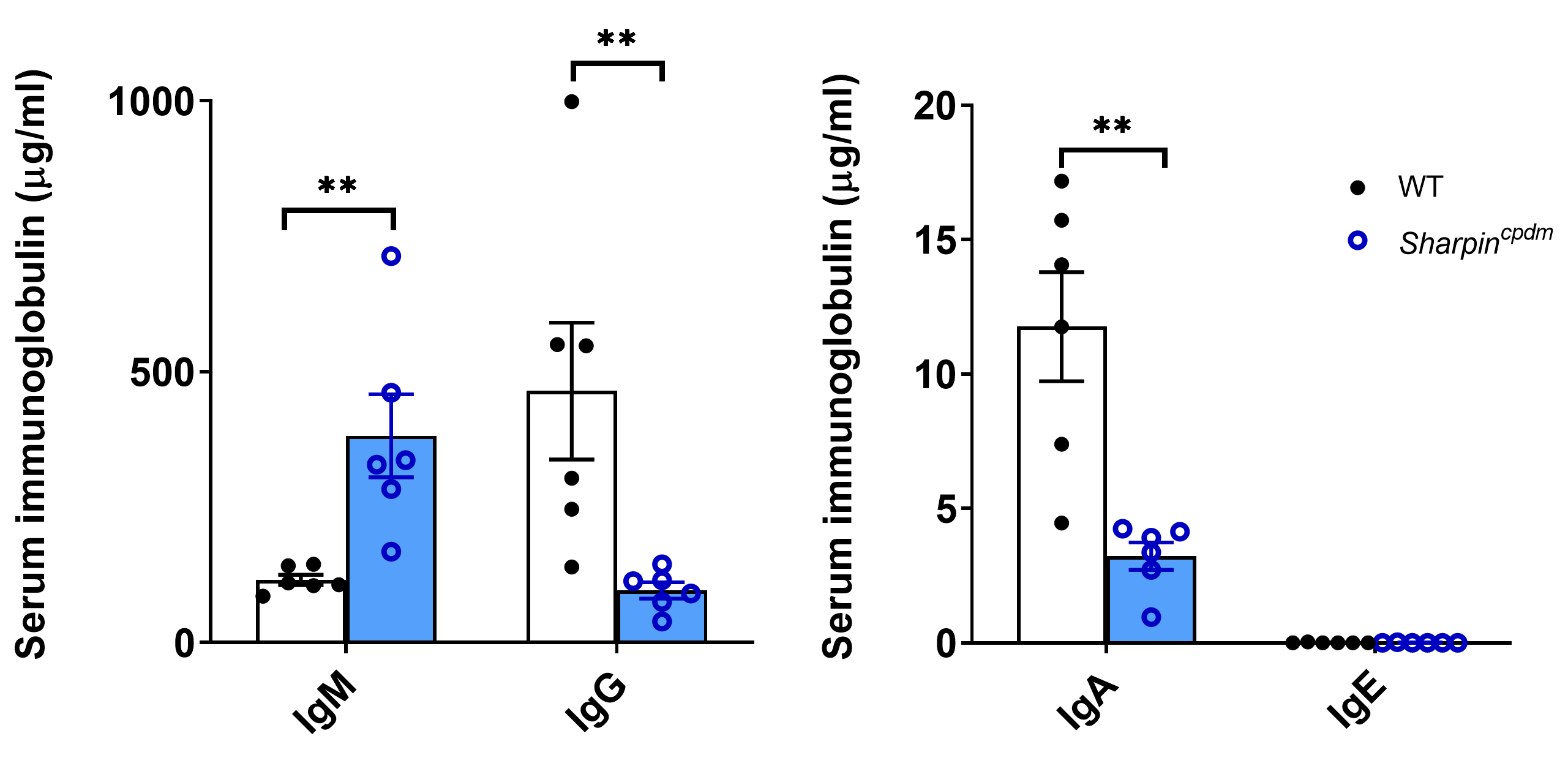

Supplement: S13 Fig — Bars represent the mean ± SE of 6 mice/group. * p < 0.05. (TIF) [file pone.0235295.s013.tif]

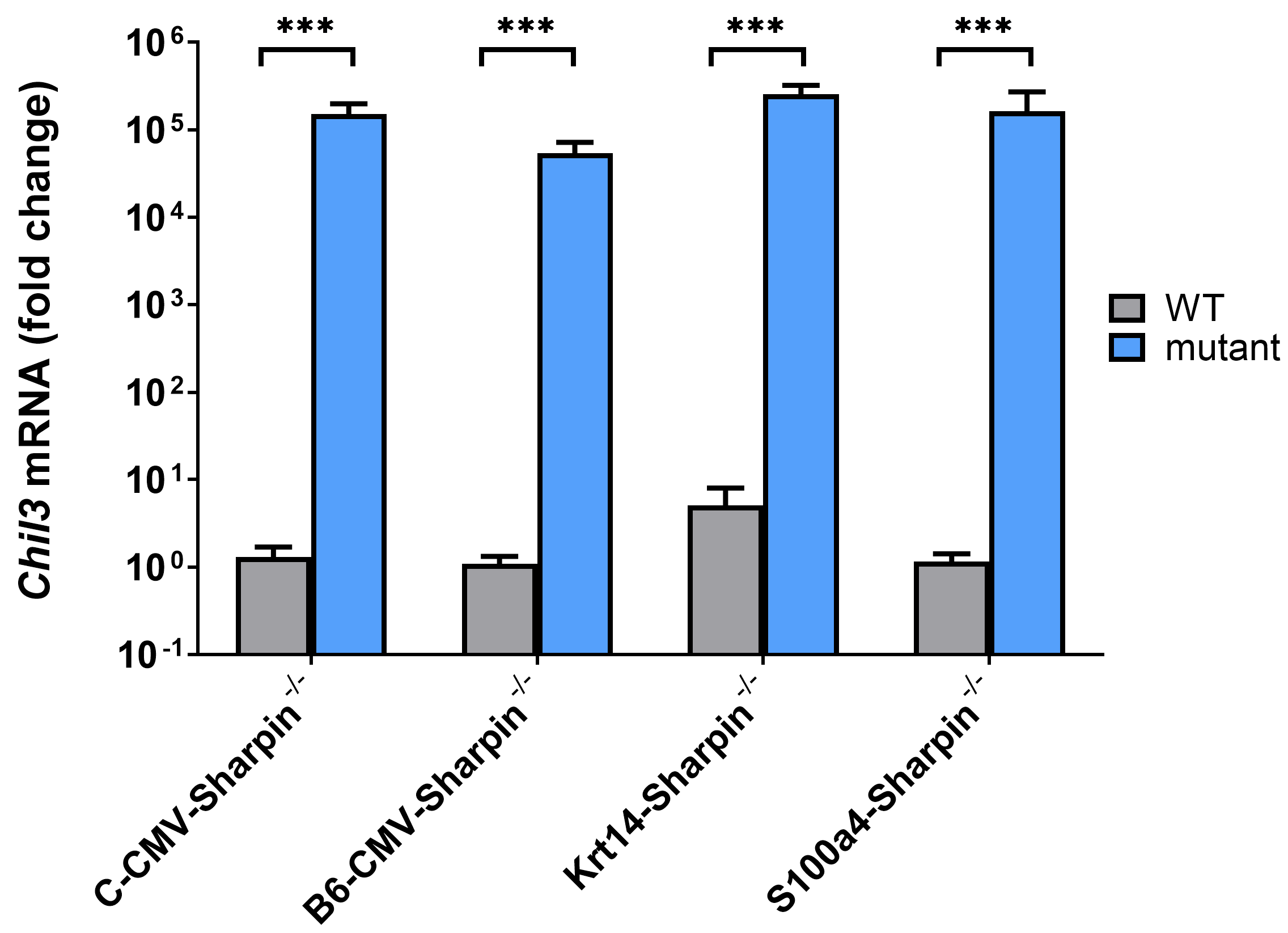

Supplement: S14 Fig — Bars represent the mean + SEM of fold change in gene expression in mutant vs. WT mice. *** P < 0.001. (TIF) [file pone.0235295.s014.tif]

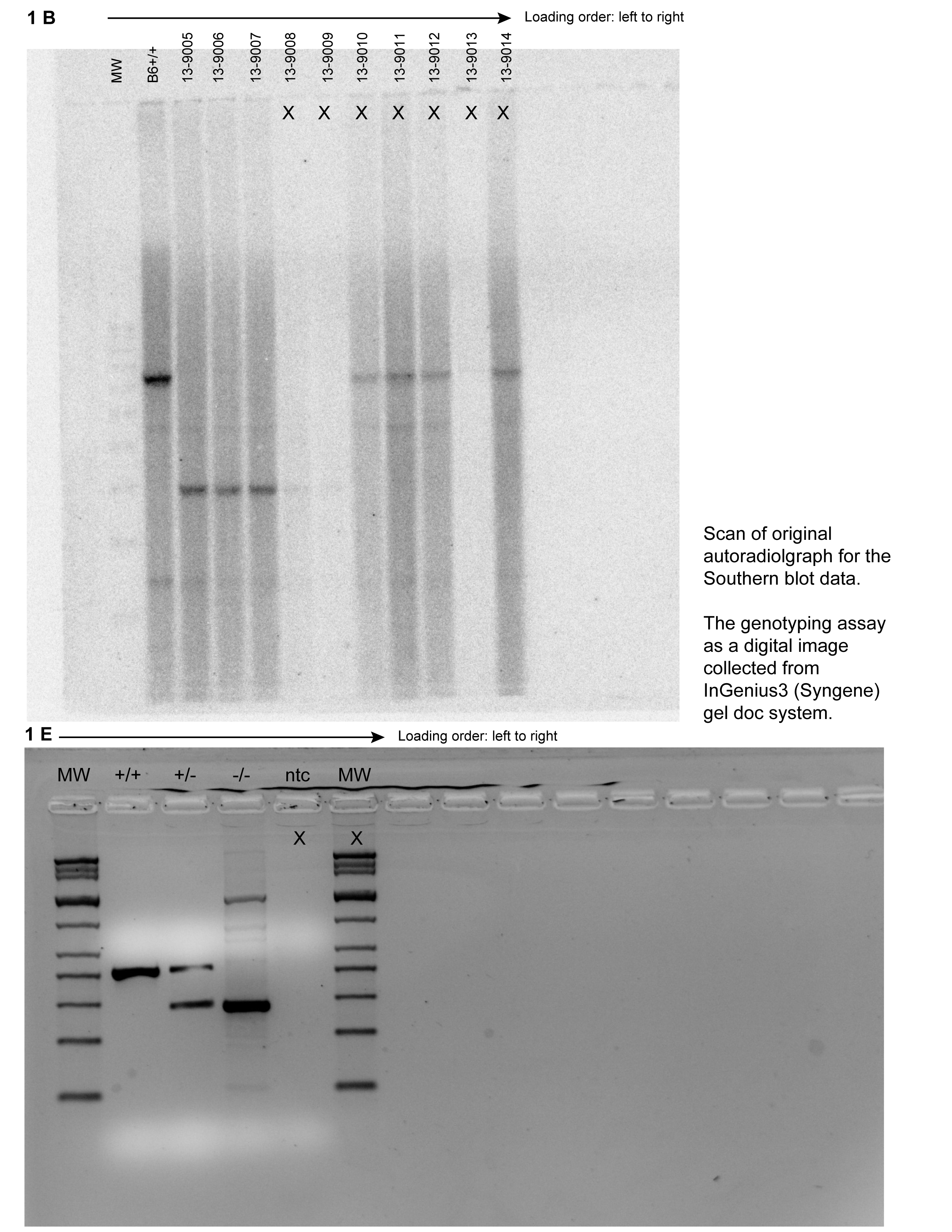

Supplement: S15 Fig — (TIF) [file pone.0235295.s015.tif]
